# Decoding c-Myc networks of cell cycle and apoptosis regulated genes in a transgenic mouse model of papillary lung adenocarcinomas

**DOI:** 10.18632/oncotarget.5035

**Published:** 2015-10-02

**Authors:** Yari Ciribilli, Prashant Singh, Reinhard Spanel, Alberto Inga, Jürgen Borlak

**Affiliations:** ^1^ Centre for Integrative Biology (CIBIO), University of Trento, 38123 Mattarello, Italy; ^2^ Centre for Pharmacology and Toxicology, Hannover Medical School, 30625 Hannover, Germany; ^3^ Institute of Pathology, 41747 Viersen, Germany

**Keywords:** c-Myc transgenic mouse model of papillary lung adenocarcinomas, whole genome scans, c-Myc regulatory gene networks, c-Myc regulated cell cycle and apoptotic genes, gene reporter assays

## Abstract

The c-Myc gene codes for a basic-helix-loop-helix-leucine zipper transcription factor protein and is reported to be frequently over-expressed in human cancers. Given that c-Myc plays an essential role in neoplastic transformation we wished to define its activity in lung cancer and therefore studied its targeted expression to respiratory epithelium in a transgenic mouse disease model. Using histological well-defined tumors, transcriptome analysis identified novel c-Myc responsive cell cycle and apoptosis genes that were validated as direct c-Myc targets using EMSA, Western blotting, gene reporter and ChIP assays.

Through computational analyses c-Myc cooperating transcription factors emerged for repressed and up-regulated genes in cancer samples, namely Klf7, Gata3, Sox18, p53 and Elf5 and Cebpα, respectively. Conversely, at promoters of genes regulated in transgenic but non-carcinomatous lung tissue enriched binding sites for c-Myc, Hbp1, Hif1 were observed. Bioinformatic analysis of tumor transcriptomic data revealed regulatory gene networks and highlighted mortalin and moesin as master regulators while gene reporter and ChIP assays in the H1299 lung cancer cell line as well as cross-examination of published ChIP-sequence data of 7 human and 2 mouse cell lines provided strong evidence for the identified genes to be c-Myc targets. The clinical significance of findings was established by evaluating expression of orthologous proteins in human lung cancer. Taken collectively, a molecular circuit for c-Myc-dependent cellular transformation was identified and the network analysis broadened the perspective for molecularly targeted therapies.

## INTRODUCTION

The molecular functions of the c-Myc oncogene have been studied in considerable detail and the seminal review of Dang [1] highlights its involvement in many biological pathways associated with neoplastic transformation, cell growth and proliferation. At its C-terminal region the c-Myc protein contains a helix-loop helix DNA binding domain and a leucine zipper dimerization motif; it functions as a heterodimeric transcription factor with its partners Max (Myc-associated x) and Mad (Max dimerization protein) at E-box (CACGTG) binding sites of targeted promoters. Subtle deviations within the canonical consensus sequence, i.e. the E-box-like motifs (CgCGTG, CACGcG or CANNTG) are also tolerated in the control of gene expression [1].

c-Myc plays a key role in cell proliferation most notable in the regulation of G1 specific cyclin dependent kinases and although almost undetectable in quiescent cells its gene transcription is rapidly induced upon mitogenic stimulation. The c-Myc protein is extraordinarily versatile and may possibly affect regulation of > 10% of entire genomes [2–4]. However, defining the molecular rules as a positive or negative regulator in the control of gene expression remain far from clear and are likely cell type and tissue specific. Moreover, as the majority of c-Myc studies are based on *in vitro* investigations, it remains uncertain which of the many c-Myc responsive genes are regulated *in vivo* and are actually responsible for the development of a given disease [5, 6].

c-Myc over-expression is detected in a large number of human cancers which inspired the development of molecularly targeted therapies [2]. There is evidence for distinct thresholds to govern c-Myc's biological activity *in vivo* whereby c-Myc's oncogenic activity may arise from its over-expression and interactions with low affinity recognition elements of otherwise c-Myc unregulated genes and by inhibition of tumor suppressor pathways [7]. Its hyper-activity may also arise without its over-expression to sustain mitogenic signaling by the repression of anti-proliferative signals and the modulation of checkpoints in the control of cell cycle regulation. Defining molecular rules by which c-Myc influences cell cycle and apoptosis regulated genes in lung cancer have not been attempted. Even so, its over-expression with or without gene amplification is common to 80–90% of small cell lung cancer (SCLC) [8]. Additionally, it was recently demonstrated that Max inactivation is able to disrupt the c-Myc-SWI/SNF network in SCLC, i.e. a cooperative gene program essential for lung cancer development [9]. c-Myc over-expression in the absence of gene amplification is also observed in about 50% of non-small cell lung cancer (NSCLC) as evidenced by immunohistochemistry of different types of NSCLC (http://www.proteinatlas.org and [8]). Furthermore, a recent study showed that the MZF1 (Myeloid Zinc Finger 1)/c-MYC axis is essential for progression of lung adenocarcinomas; it is mediated by the cellular loss of the wild-type liver kinase B1 gene, i.e. a tumor suppressor frequently repressed in the pathogenesis of epithelial cancers [10].

Specifically, lung cancer is one of the most common cancers in terms of incidence and mortality, with NSCLC contributing to the bulk of disease burden [11]. Several genetic events are necessary for malignant transformation of respiratory epithelium and include over-expression and/or mutation of proto-oncogenes as well as loss of tumor suppressor functions [12]. It was shown earlier that patients with Caspase-3 positive and c-Myc negative tumors had a better prognosis as compared to patients with Caspase-3 negative and c-Myc positive tumors [13]. Moreover, c-Myc was reported to foster metastasis in an animal model of NSCLC [14]. Recently it was demonstrated that c-Myc cooperates with mutation-activated BRAF^V600E^ during mouse lung cancer development by suppressing senescence [15] and was shown to promote tumor aggressiveness in non-small cell lung cancer through suppression of miRNA-29b [16].

Altogether, c-Myc is frequently elevated in tumors and genetic alterations such as translocations, gene amplifications and mutations in regulators of c-*myc* expression directly affect c-Myc activity, nonetheless the molecular pathology of organ specific tumors differ to suggest cell type and tissue specific gene regulatory networks. For instance different studies analyzed genome wide the impact of increased c-Myc levels in human cell lines derived from multiple myeloma, SCLC and glioblastoma multiforme [17] or osteosarcoma and cervix carcinoma [18] as well as from murine lymphoma mouse models [19] and primary lymphocytes in addition to embryonic stem cells [20].

In an effort to define genetic events associated with c-Myc transforming capacity in lung cancer a transgenic disease model of NSCLC/papillary adenocarcinomas was studied and we focused on cell cycle and apoptosis regulated genes in response to oncogenic c-Myc signaling.

## RESULTS

**Figure 1:**
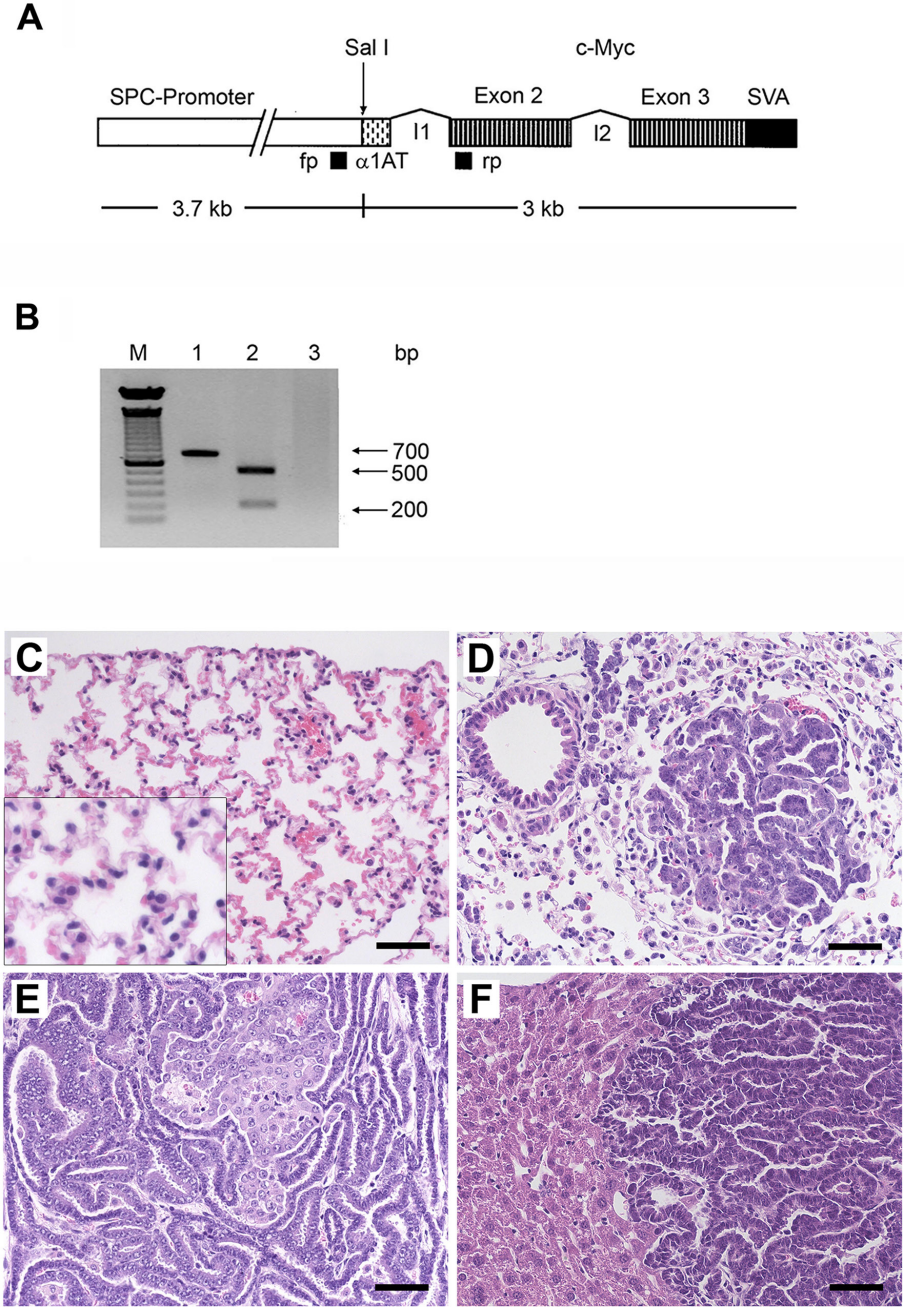
Histopathology of lung cancer and c-Myc transgene verification by PCR **A.** Scheme of the transgene for the production of transgenic mice. SPC promoter: human surfactant protein promoter; α1AT: first exon of the non-coding alpha 1 antitrypsin gene; I1: intron 1 of the alpha 1 antitrypsin gene fused to the first intron of the c-myc proto-oncogene; I2: intron 2 of the c-myc proto-oncogene; SVA: SV40 Poly A signal. The primer binding sites used for an identification of the transgene are indicated by black boxes: fp, forward primer; rp, reverse primer. **B.** Myc-transgene PCR of tail biopsy DNA. Lane 1–2: transgenic mice; lane 2: The amplified DNA was digested with Sal 1 to obtain fragments of about 200 and 500 bp; lane 3: non-transgenic controls; M, molecular weight standard. **C.** Normal subpleural parenchyma of non-transgenic controls. The insert represents a 2-fold magnification and depicted are normal pneumocytes with small regular nuclei. **D.** Illustrated is an initial papillary lung adenocarcinoma (PLAC) with real papilla, its own stroma and a size of 220 μm in diameter. Around the tumor numerous foci of dispersed AHH of the BAC-type are seen. **E.** Advanced PLAC with folded papillary structures of secondary and tertiary degree. **F.** Liver metastasis of PLAC. The bar represents 50 μm.

A scheme of the gene construct for the production of transgenic mice is given in Figure 1A. The presence of the transgene was verified by PCR and agarose gel electrophoresis of tail biopsy DNA (Figure 1B).

### Histopathology of lung tumors

Lung tissue from control non-transgenic and of transgenic mice was examined. At the age of about 6–7 month transgenic animals were characterized by an alveolar lining of dysplastic epithelium with no evidence for basement membrane fragmentation. Hence, the observed dysplasia was considered to be an epithelial precursor lesion. At later stages (9–13 month) medium-sized or large solid tumors were observed, some of which consumed an entire lobe, while the remaining parenchyma appeared to be macroscopically unaffected. Nonetheless, ubiquitous proliferations of the peripheral pulmonary epithelium were evident at the microscopic level (Figure 1C and 1D). The alveoli and parts of the terminal bronchioli were occupied by numerous foci of hyper-chromatic low columnar cells, thus evidencing the classic lepidic growth pattern. These foci were dispersed throughout the whole lung and eventually formed a confluent monolayer within the alveoli. The lesional cells displayed basophilic cytoplasm and marked nuclear atypia, as defined by vesicular enlargement and prominent nucleoli, however, exhibited only slight nuclear polymorphism.

Histologically, the lesions were defined as non-invasive precancerous lesions and were considered similar to atypical adenomatous hyperplasia (AHH) as defined by the current human lung tumor classification.

Mitosis was repeatedly seen, e.g. at least 1 mitosis/high-power field (= HPF) in confluent lesions (data not shown). The macroscopically visible solid tumors and the microscopically observed initial tumors were invasively growing carcinomas, all invariably with a papillary growth pattern (Figure 1D and 1E). While initial papillary lung adenocarcinomas (PLAC) were minimally invasive (Figure 1D), advanced PLAC developed the whole range of malignancy as defined by the gross bronchial and vascular invasion and metastatic spread including that of liver metastasis as depicted in Figure 1F.

Macroscopically, solitary tumors of different sizes were mostly noted. Microscopically, for a given lobe up to five individual tumor foci were counted; an entire lung may present up to 25 multi-centric invasive tumors.

### Genome-wide transcript expression profiling

**Figure 2:**
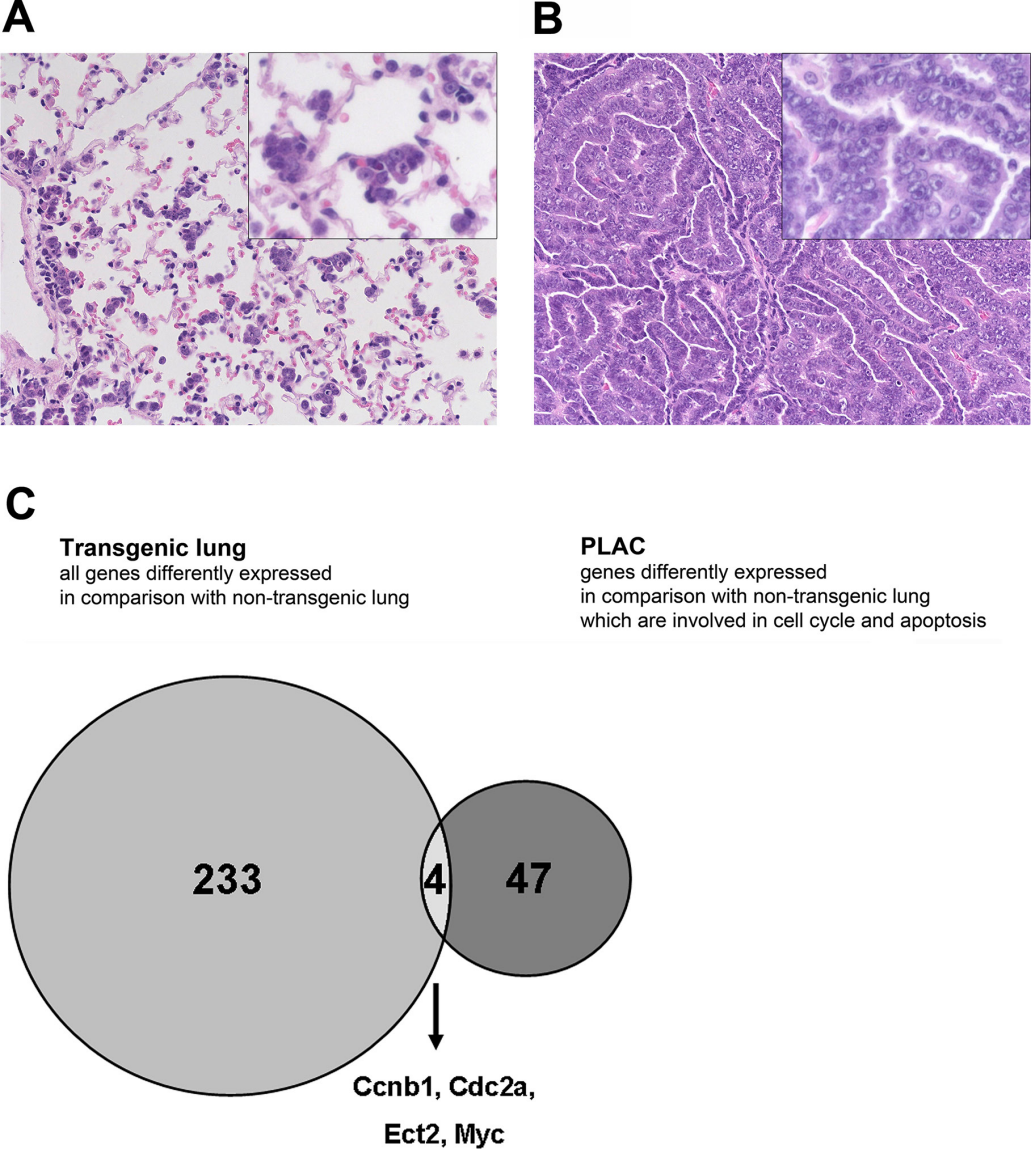
Differentially expressed genes in c-Myc transgenic lung and lung tumors **A.** Histology of transgenic lung with pre-cancerous lesions (AHH of BAC-type). The insert represents a 2-fold magnification and depicted are groups of atypical bronchioloalveolar cells with enlarged vesicular nuclei. **B.** Papillary lung adenocarcinoma (PLAC). The insert represents a 2-fold magnification. Depicted are high columnar tumor cells with vesicular nuclei and prominent nucleoli. When compared to the atypical bronchioloalveolar cells of AHH the nuclei in PLAC are further enlarged. **C.** Venn Diagram: Number of regulated genes in transgenic lung and c-Myc regulated cell cycle and apoptosis genes in invasive PLAC. Note, only 4 genes are commonly regulated in transgenic lung and PLAC to suggest different activities of wild type and oncogenic c-Myc.

In an effort to identify novel candidate MYC target genes and to confirm their tumor relevant regulations whole genome gene expression data obtained from lung tissue of wild type, non-transgenic control animals were compared to lung tumors of transgenic mice (see Supplementary Figure S1 for the strategy of data analysis). Statistical analysis of wild type control and lung tumors/PLACs identified 162 genes with significantly increased expression (mean FC > 3, *p*-value in *T*-test < 0.05 and 100% of “Increase” calls in comparative ranking analyses in one or more sets of tumors); however, about twice as many genes (*n* = 301) were repressed (FC < −3, *p*-value in *T*-test < 0.05, and 100% “Decrease” calls accordingly) (Table 1). Subsequently, the tumor data (162 + 301 = 463) were filtered for cell cycle and apoptosis genes using the criteria described in the Materials and Methods section; this defined 51 significantly regulated genes of which 27 were up- and 24 were down regulated (see Supplementary Table S1, Figure 1E & 2B depicting PLAC). To delineate candidate MYC target genes and to confirm their tumor relevant regulations whole genome gene expression data from transgenic non-carcinomatous lung were compared to healthy non-transgenic lung and such comparison yielded a total of 237 genes (Figure 2C, Supplementary Table S10). Next, the data were compared to transgenic lung tumors filtered for cell cycle and apoptosis genes to eventually define 47 uniquely and 4 common regulated genes. As depicted in Figure 2A the histology of transgenic lungs clearly demonstrated the absence of carcinomatous lesions and in such transgenic lungs a moderate 3-fold increase in c-Myc gene expression was observed. Importantly, with the exception of c-Myc, the cyclin dependent kinase A-1, the G2 mitotic specific cyclin B1 and the transcription factor ect2 none of the regulated genes in transgenic lungs were regulated in lung tumors thus providing evidence for their regulation by constitutive but not oncogenic c-Myc (Figure 2C, Supplementary Table S1).

**Table 1:** Statistics of differentially expressed genes in SPC/c-Myc-transgenic lung tumors

UP-regulated genes		Number of genes containing c-Myc binding sites	
all up-regulated genes in tumors	**162**	**67**	**41,4%**
Known direct c-Myc-targets (**T**)	32	20	62,5%
relatives of known direct c-Myc-targets (**rT**)	15	5	33,3%
Known c-Myc-responsive genes (**R**)	16	8	50%
relatives of known c-Myc-responsive genes (**rR**)	9	2	22,2%
new c-Myc-responsive genes	90	32	35,6%

DOWN-regulated genes		Number of genes containing c-Myc binding sites in their promoters	
all down-regulated genes in tumors	**301**	**71**	**23,6%**
Known direct c-Myc-targets (**T**)	5	0	0%
relatives of known direct c-Myc-targets (**rT**)	16	3	18,8%
Known c- Myc- responsive genes (**R**)	24	6	25,0%
relatives of known c-Myc-responsive genes (**rR**)	17	4	23,5%
new c-Myc responsive genes	239	58	24,3%

A total of 5269 and 4706 genes were expressed in control lung (*N*=4) and tumors (*N*=10) using the criteria signal value ≥70 and “present” detection call in Affymetrix microarray analysis. Differentially expressed genes are defined by a fold change ≥3 or ≤-3 and 100% concordance in increase or decrease change calls of tumor samples, respectively. Statistical significance was considered in *T*-test analysis between sets of small (*N*=3), middle (*N*=4) and large size tumors (*N*=4) at *p*<0,05.

In strong contrast, gene expression profiling of solid tumors (see Figure 1E & 2B depicting PLAC) defined genes targeted by oncogenic c-Myc with c-Myc amplification being increased up to > 58-fold (Supplementary Table S1). However, the gene expression of the *c-myc* heterodimeric partner Max was only minimally increased and the gene coding for *mad4* was expressed alike in all lung tissues analyzed; note *mad4* competes for Max binding to repress c-Myc activity. Moreover, expression of Miz-1, a mediator of c-Myc dependent gene repression [21] did not differ between healthy lung tissue from control animals and tumors.

**Figure 3:**
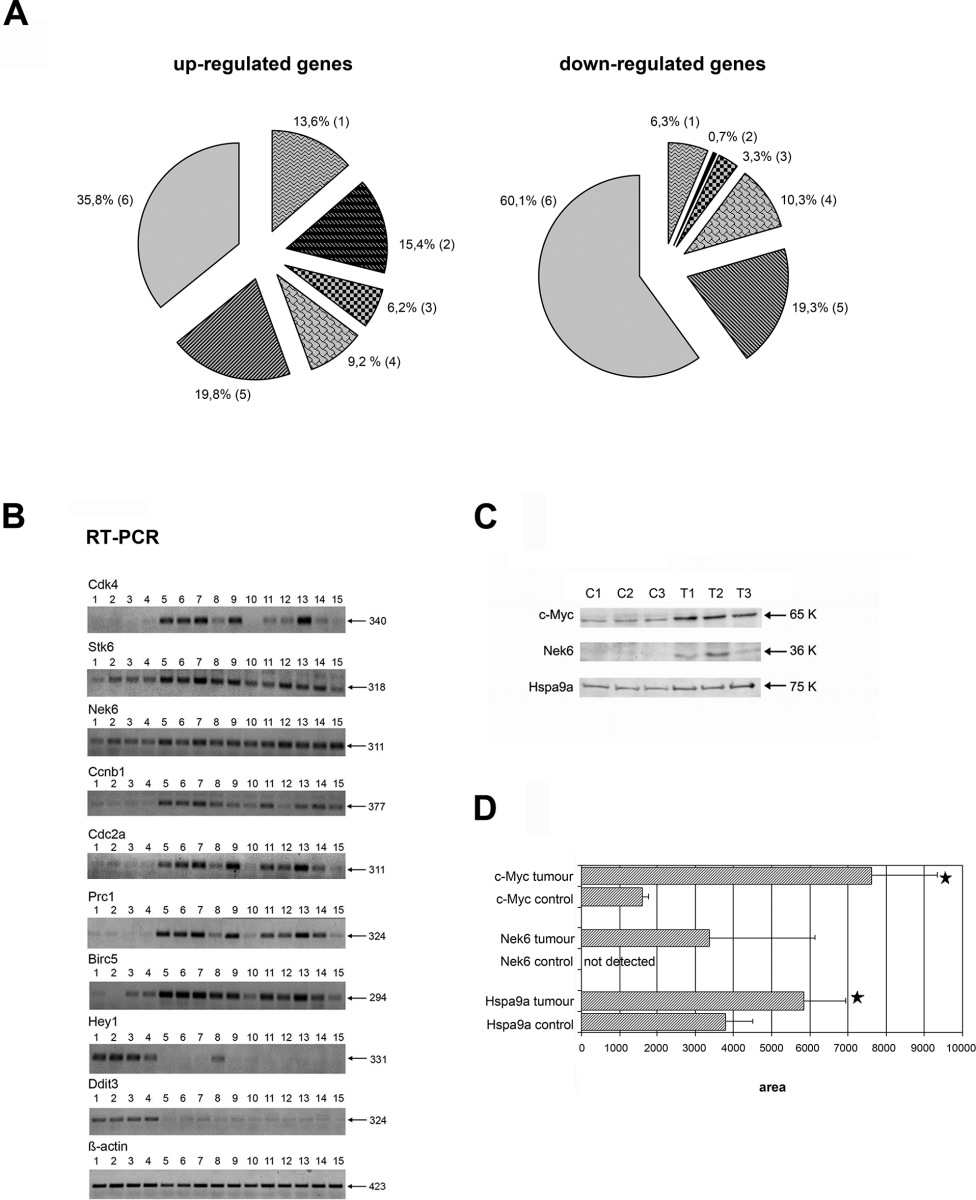
Summary information of differentially expressed genes in c-Myc lung cancer and validation of candidate genes by RT-PCR and Western blotting **A.** Differentially expressed genes in lung adenocarcinomas of c-Myc-transgenic mice: known responsiveness/interaction with c-Myc; proportion (%) of genes containing potential c-Myc binding sites in the promoter region. Left panel: results for 162 up-regulated genes; Right panel: results for 301 down-regulated genes. 1 - known c-Myc targets and their relatives. 2 - known c-Myc targets and their relatives containing c-Myc binding sites. 3 - known c-Myc-responsive genes and their relatives containing c-Myc binding sites. 4 - known c-Myc-responsive genes and relatives. 5 - new c-Myc-responsive genes with predicted and experimentally proven c-Myc binding sites. 6 - new c-Myc-responsive genes. **B.** RT-PCRs for selected genes: lanes 1–4: control lung; lanes 5–7: pools of small-sized tumors; lanes 8–12: middle-sized tumors; lanes 13–15: large-size tumors. **C.** Western blot analysis for selected genes: C1–C3 - control non-transgenic lung; T1–T3 - papillary lung adenocarcinomas of SPC/c-Myc-transgenic mice. **D.** Densitometric scans of Western blots; bars marked with a * are significantly different from non-transgenic animals; *p* < 0.05.

Differentially expressed genes (DEGs) were also compared to published data where c-Myc target genes were listed [22–24]. Such comparisons revealed known c-Myc-responsive genes, i.e. genes whose expression was changed in response to c-Myc activation, as well as known c-Myc targets, e.g. genes known to bind c-Myc directly (Table 1 and Supplementary Table S1). Out of 162 genes up-regulated in tumors, 29% were already reported as c-Myc targets or their relatives, i.e. those belonging to gene families which include known c-Myc targets (Figure 3A, left panel). Many of these genes code for proteins involved in cell cycle regulation (e.g. Cdk4, Ccnb1 and Cks2) and apoptosis (e.g. Hspa9a and Trp53). Alike, among the 301 repressed genes 7% are known c-Myc targets or their relatives (Table 1, Figure 3A, right panel); nonetheless about half of the genes with increased expression in PLAC (56%) and the majority of down-regulated genes (79.4%) are so far unknown as c-Myc-targets in lung cancer. Taken collectively, new c-Myc-target and responsive genes were identified and included over-expressed Stk6, Nek6, Prc1, Ect2, and Birc5 and repressed Cdkn2d, Lats2, Bnip2 and Hey1, which are highly interesting disease candidate genes for their essential role in the regulation of cell cycle and programmed cell death. The expression of some genes known to be c-Myc-responsive such as CEBPα was oppositely regulated in lung adenocarcinomas when compared with previously reported studies, therefore pointing to tissue-specific responses to c-Myc activity. These genes are *italicized* in Supplementary Table S1A and S1B. Note, for its known c-Myc responsiveness, cyclin D1 was included in Supplementary Table S1 and even though the fold change was significant it did not meet the set threshold criteria of > 3-fold. In Supplementary Table S1 the common regulated genes, i.e. *c-myc*, *ccnb1*, *cdc2A* and *ect2* are shown in bold and were included as their expression increased significantly from transgenic lung tissue to tumor.

In regards to tumor size no qualitative difference in gene expression profiles was observed. This agrees with the histological examination of tumors all of which were classified as PLACs of various sizes. However, some quantitative differences were observed (up to 2- to 3-fold) in the expression level of some genes between small and large tumors. Among those with substantially higher expression level in small-size tumors were cell cycle regulators and the proto-oncogene ect2. Furthermore, expression of some genes in tumors were absent, while their expression was abundant in non-transgenic healthy control lungs (highlighted by grey rows accordingly in Supplementary Table S1B). This included the pro-apoptotic Ddit3 and transcription factor Nr2f1, which exerts anti-AP-1 activity and are a mediator of the anti-cancer effect of retinoic acid [25].

### Validation of microarray data by RT-PCR and Western blotting of regulated genes

To verify results by an independent method RT-PCR assays were performed by selecting several up-regulated cell cycle and apoptosis genes, i.e. Cdk4, Stk6, Nek6, Ccnb1, Cdc2a, Prc1 and Birc5 as well as the down-regulated (bHLH)-type transcriptional repressor Hey1 and the multifunctional transcription factor Ddit3. The changes in gene expression found in lung tumors by both methods (microarray & RT-PCR) were in agreement (Figure 3B, Table 2). In addition, the regulation of Cdk4, Ccnb1, Cdc2a, Prc1 and Birc5, which are part of the mortalin network (see below), was supported by RT-PCR assays. Besides, c-Myc and Nek6 protein expression was evaluated by Western blot analysis (Figure 3C), and quantified by gel densitometry (Figure 3D) to confirm their regulation at the protein level.

**Table 2:** Validation of microarray data by RT-PCR

Gene	Method	Mean FC size of tumors		
		small	middle	large
Cdk4	RT-PCR	3.0	2.0	1.4
	Affymetrix	2.7	2.1	2.9
Stk6	RT-PCR	6.6	3.1	4.1
	Affymetrix	6.4	3.4	4.2
Nek6	RT-PCR	3.0	2.6	2.9
	Affymetrix	3.1	3.2	3.5
Ccnb1	RT-PCR	9.5	4.0	5.4
	Affymetrix	22.2	9.4	12.8
Cdc2a	RT-PCR	13.3	6.5	8.3
	Affymetrix	18.0	10.6	10.5
Prc1	RT-PCR	8.0	5.1	5.5
	Affymetrix	6.0	3.0	4.2
Birc5	RT-PCR	10.1	4.8	4.9
	Affymetrix	8.6	4.5	6.0
Hey1	RT-PCR	A	−1.9	A
	Affymetrix	−4,9	−3.7	−5,1
Ddit3	RT-PCR	−4.0	−3.5	−5.9
	Affymetrix	−2,3	−3,5	−6,2

A - “Absent” - no gene expression was detected

The gene expression values in all samples were normalized to the beta-actin expression and used to calculate mean expression values for tumors of various sizes and control lung tissue extracts. The mean fold change was computed as a ratio between mean gene expression values for tumor sets and control non-transgenic lung as a baseline.

### Search for c-Myc gene networks

A bioinformatics strategy to search for regulatory gene networks in lung adenocarcinomas of c-Myc transgenic mice was previously published [26]. In the present study 8 different position weight matrices (PWMs) containing E-box motifs were applied to genomic sequences of regulated genes (see Materials and Methods section for details and Supplementary Figure S2). Among genes regulated in tumor and non-tumorous transgenic lung tissue (see Supplementary Tables S1 and S10 for transgenic non-tumor data) 24, 22 and 198 c-Myc binding sites were identified (Supplementary Table S11). The average number of c-Myc binding sites in promoters of up-regulated genes was increased by 2-fold when compared to repressed genes and included the known c-Myc targets Cdk4, Kif11, Hspa9a, Aurka or Stk6, Ccnb1, Trp53 and Cebpα as well as the putative gene targets Prc1, Elf5, Klf7, Lats2 and Anp32a (Supplementary Table S1). Repression of several transcription factors was observed, some carrying c-Myc binding sites in their promoters such as Foxf1, Tbx3 and Klf7 (Supplementary Table S1).

**Figure 4:**
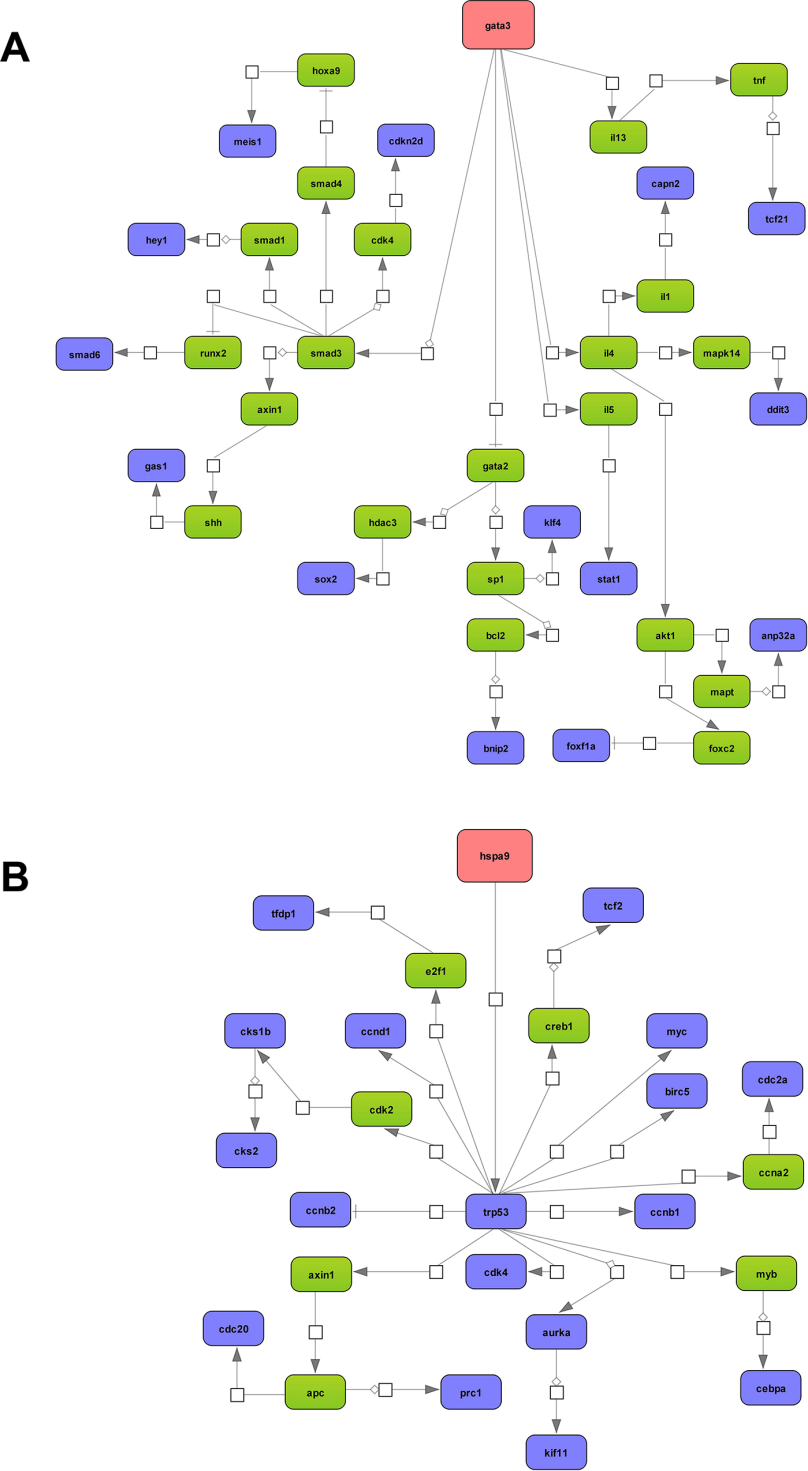
Master regulatory gene networks in lung tumors of c-Myc transgenic mice **A.** Gata 3 master regulatory network of repressed gene expression. 63% or 15 out of 24 down- regulated genes are part of the GATA3 regulatory network. **B.** Hspa9 (= mortalin) master regulatory network of induced gene expression. 67% or 18 out of 27 regulated genes are part of the Hspa9 network. The networks were constructed with the GeneXplain platform; the colour coding red, blue and green represent nodes for master regulator, regulated genes and connecting genes, respectively. An activation, inactivation and regulation are denoted by the symbols 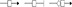, respectively.

Next, master regulatory molecules were defined by utilizing the GeneWays network information and a maximum radius of 4 steps upstream of an input dataset was selected. In the case of tumor associated repressed genes the analysis revealed Gata3 as master regulator and 63% or 15 out of 24 are part of the regulatory gene network (Figure 4A). The entire network consisted of 36 genes, however *n* = 21 remained unchanged in expression. Note Gata3 is part of the composite module that defines repressed gene expression and the gene coding for this zinc finger protein was highly significantly repressed to 15% of control values in the lung tumors analyzed. Gata3 plays a key role in airway remodeling during organ development [27] and this transcription factor functions as a tumor suppressor by controlling the expression of lung metastasis inhibitors (DLC1 (deleted in liver cancer 1) and PAEP (progestagen-associated endometrial protein) [28]. Cooperativity between Myc and Gata3 in the transcriptional control of gene expression was also reported [29].

Employing the same strategy Hspa9 (=mortalin) was identified as master regulator for tumor up-regulated genes. This protein is a member of the Hsp70 heat shock protein (HSP) family and functions as a molecular chaperone; it was up-regulated by 3.3 fold in the tumors analyzed and 18 out of 27 up-regulated genes are part of the Hspa9 network (Figure 4B). c-Myc binding to Hspa9 promoter sites is supported by EMSA (see below) and the targeting of Hsp70 and Hsp90 in the treatment of lung cancer is actively pursued.

**Figure 5:**
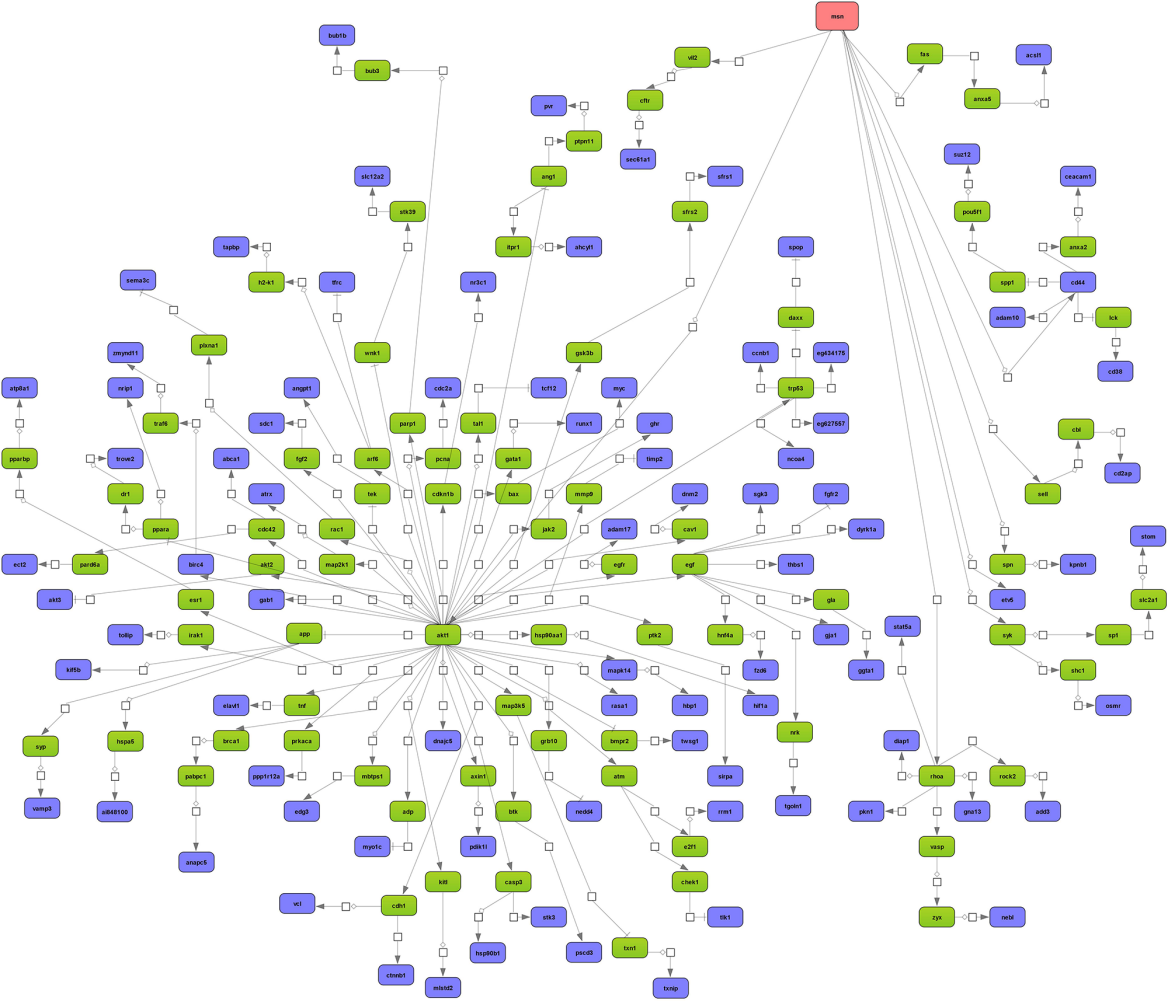
Master regulatory gene networks in non-tumor c-Myc transgenic mice Depicted is the moesin master regulatory network. Nearly 38% or 88 out of 233 of the up-regulated genes contributed to this network. The networks were constructed with the GeneXplain platform. The color coding red, blue and green represent nodes for master regulator, regulated genes and connecting genes, respectively. An activation, inactivation and regulation are denoted by the symbols 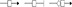, respectively.

In the same way the non-tumor transgenic data were analyzed. Here, Msn (=moesin) was defined as a master regulator and contributed to 52% (88 out of 171) of the overall network (Figure 5). Msn itself was up-regulated by nearly 4-fold and the protein links the actin-cytoskeleton to the plasma-membrane [30] to function as a tumor suppressor in lung cancer

### Functional composite modules

**Figure 6:**
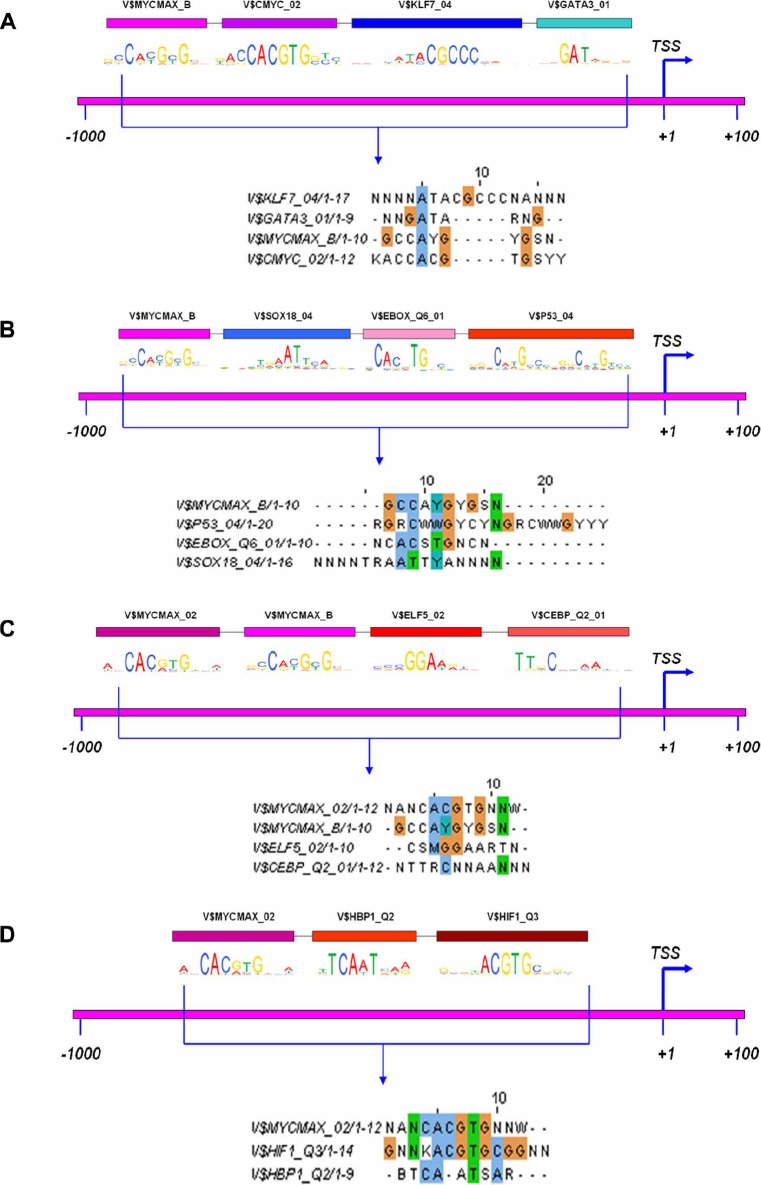
Functional transcription factor composite modules The co-occupancy of different transcription factor binding sites at gene specific promoters was analyzed. The promoter region with the best possible composite module is depicted with the consensus motif sequence locus. The software Clustal W2 (http://www.ebi.ac.uk/Tools/msa/clustalw2/) was used for multiple sequence alignment (MSA) of motifs as to define overlapping regions. **A.** Composite module for down-regulated genes. 63, 67 and 96% had consensus binding sites for c-Myc, Klf7 and Gata3, respectively; the entire composite module fitted 46% of regulated genes. **B.** Alternate composite module for down-regulated genes. 92, 92 and 96% had consensus binding sites for c-Myc, Sox18 and P53, respectively; the entire composite module fitted 83% of regulated genes. **C.** Composite module for up-regulated tumor genes. 74, 96 and 93% had consensus binding sites for Myc, Elf5 and Cebpα, respectively; the entire composite module fitted 63% of up-regulated genes. **D.** Composite module for up-regulated non-tumor transgenic lung. 43, 25 and 53% genes had consensus binding sites for c-Myc, Hbp1 and Hif1, respectively.

The co-occupancy of different transcription factors was analyzed at gene specific promoters and for tumor regulated repressed genes three composite modules were defined by the genetic algorithm (Supplementary Table S5). Two of them consisted of Myc, Klf7 and Gata3 or Myc, Sox18 and P53. The genes coding for these transcription factors were regulated as well (see Supplementary Table S1) and the composite module comprising Klf7 and Gata3 was considered to be highly relevant as both TFs were repressed in expression (see Supplementary Table S1 and Figure 6A). Among 24 down-regulated genes 15 (63%), 16 (67%) and 23 (96%) had consensus binding sites for c-Myc, Klf7 and Gata3, respectively while the entire composite module fitted 11 promoters or 46% of down-regulated tumor specific genes. Likewise, 22 (92%), 22 (92%) and 23 (96%) genes contained binding sites for c-Myc, Sox18 and P53, respectively while the entire composite module fitted 20 promoters or 83% of down-regulated genes (Figure 6B). Besides, c-Myc DNA binding at Klf7 and P53 gene specific promoter sites were confirmed by EMSA band shift assay as detailed below. It was shown earlier that p53 expression is induced by c-Myc in NSCLC cells [31]. Conversely, repressed expression of Sox18 in lung tumors of c-Myc transgenic mice may be caused by hyper-methylation, i.e. an epigenetic mechanism [32]. In the case of tumor specific up-regulated genes the predicted composite module comprised of Myc, Elf5 and Cebpα binding sites found in 20 (74%), 26 (96%) and 25 (93%) genes, respectively while the entire composite module fitted 17 promoters or 63% of up-regulated genes (Figure 6C). Likewise, c-Myc binding at gene specific promoter sequences of Elf5 and Cebpα was confirmed by EMSA band shift assay as detailed below. Lastly, a composite module (score: 47.63) for up-regulated DEGs in non-tumor transgenic lung was computed and consisted of Myc, Hbp1 and Hif1 (Figure 6D). Binding sites for these transcription factors were found in 101 (43%), 58 (25%) and 124 (53%) genes, respectively. It was shown earlier that Hbp1 functions as a tumor suppressor [33] by inhibiting oncogenic Wnt-β-catenin signaling [34] while oncogenic c-Myc activity is inhibited by its interaction with the tumor suppressor protein Hbp1 [35]. In non-cancerous transgenic lungs the gene expression of Hbp1 and Hif1 was up-regulated by 3 and 5-fold, however was unchanged in lung tumors.

### Experimental validation of gene networks by electrophoretic mobility shift assay

**Figure 7:**
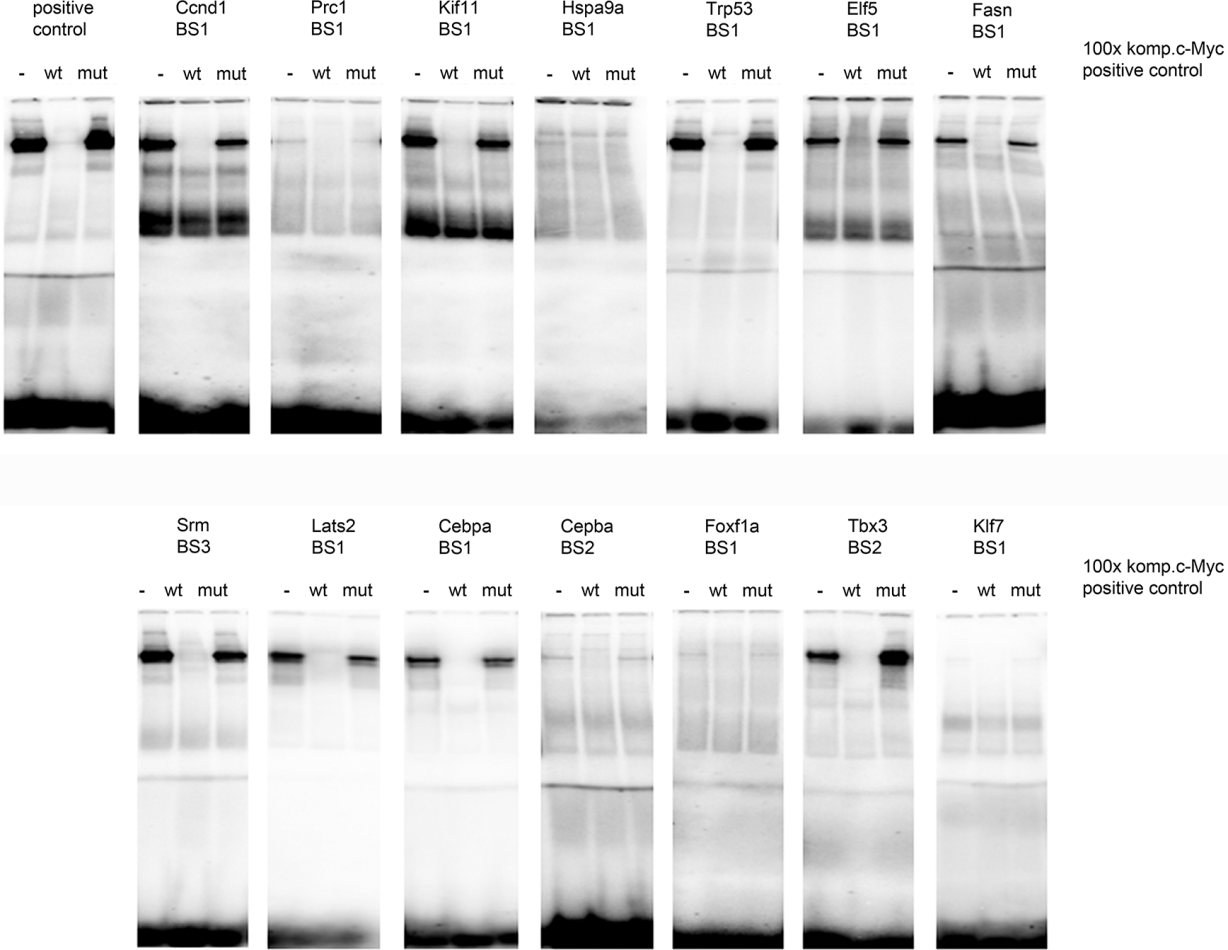
c-Myc DNA binding activity at gene specific promoters c-Myc DNA-binding at predicted gene-specific promoter sites of candidate genes was determined by EMSA. A total of 14 c-Myc binding sites were investigated using oligonucleotide probes described in Supplementary Table S8. Nuclear extracts from HeLa cells served as positive control (= positive control). DNA binding activity was assayed using probes specifically designed to recognize the predicted consensus binding site and is marked as – in the first lane of each gel. Specificity of DNA binding activity was determined in competition assays using 100-fold excess of the unlabeled probe (marked as wt = wild type) and by using a mutated (mut) probe whose nucleotide sequence was altered to be unable to recognize the core consensus binding site. Next to the positive control DNA binding activity was confirmed for Ccnd1, Prc1, Kif11, Trp53, Elf5, Fasn, Srm, Lats2, Cebpa, Foxf1 and Tbx3 and the observed bands were removed in competition assays with excess unlabelled probe (× 100 − fold) but not with the mutated probes. Notably, several commercially available C- and N- terminus directed antibodies were tested for their use in band shift assays but none of tested antibodies proved useful.

c-Myc DNA-binding activity was studied at predicted promoter sites by EMSA; its binding activity was lost in competition assays at 100-fold excess of unlabeled probe. Probes mutated by a single base in the E-box motif were unable to shift the bands therefore demonstrating specificity. A total of 14 Myc binding sites at 13 gene specific promoter sites were studied (see Figure 7 and Supplementary Table S8 for full description of the binding sites). Next to the positive control c-Myc DNA binding activity was confirmed for 11 selected genes. Unfortunately, the yield of nuclear extracts from lung tumors was too low to carry out EMSA assays. Therefore, assays were performed with nuclear extracts from liver tissue of another transgenic mouse model with targeted expression of c-Myc to the liver. These mice develop cancer as well [36] and are a rich source for nuclear proteins. In addition nuclear extracts of HeLa cells served as a positive control. Amongst the tumor specific genes Gata3 was of great importance. This tumor suppressor functions as a master regulatory molecule in the network of repressed genes, and, consistently, its transcript expression was repressed in lung tumor samples. Together with Gata3 the transcription factor Klf7 is part of the composite module (Figure 6A) and predicted to be a target of c-Myc. However, c-Myc DNA binding activity at one of the chosen Klf7 promoter sites was minimal and likely non-significant (Figure 7). Although c-Myc DNA binding activity was minimal at one of the 3 predicted Foxf1a promoter sites its transcript expression was significantly up-regulated. Note, this protein is part of the GATA3 gene network. Strong c-Myc DNA binding activity was determined for one of the two promoter sites of the Cebpα promoter and this factor is part of the composite module that defines the up-regulated tumor specific gene expression dataset (Figure 6C). EMSA assays also revealed weak DNA binding activity at one of the several c-Myc binding sites in the Hspa9 promoter sites and this protein functions as a master regulator for tumor specific up-regulated genes. Lastly, and with the exception of Prc1 strong c-Myc DNA binding activity was evidenced for Ccnd1, P53 (Trp53 in Figure 7) and are part of the mortalin master regulatory network.

### ChIP assays with the human lung adenocarcinoma cell lines A549 and H1299

**Figure 8:**
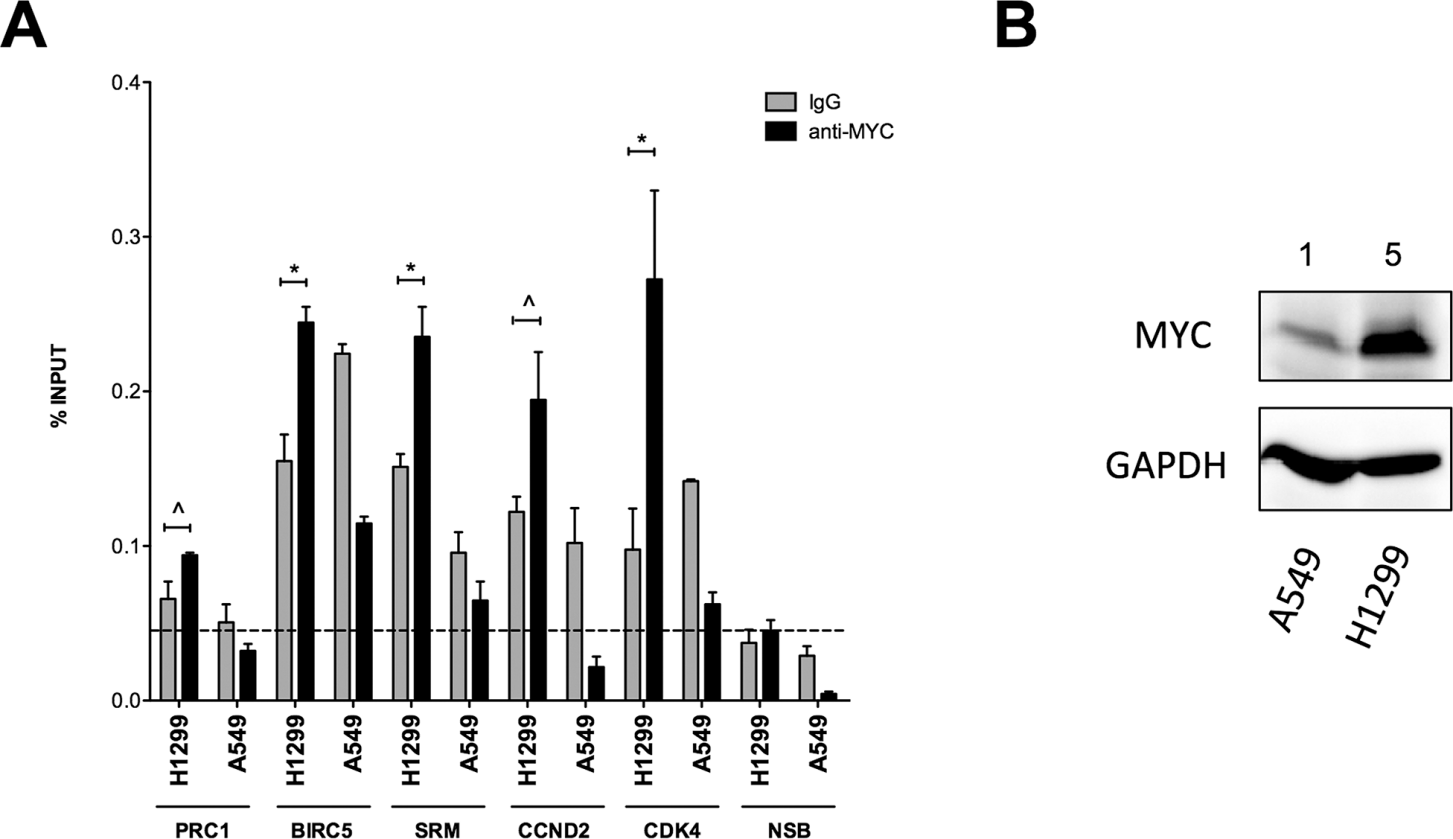
Occupancy analysis of newly established MYC target genes **A.** qPCR quantification of immuno-precipitated DNA fragments from H1299 and A549 Non-Small Cell Lung Cancer (NSCLC)-derived cell lines. Chromatin sheared sections samples were subjected to overnight incubation with anti-MYC monoclonal antibody (black bars) or mouse normal IgG (grey bars). Enriched DNA fragments were analyzed by qPCR using primers specifically located in the promoter regions of putative MYC target genes. CCND2 and CDK4 served as positive control and two loci within CCNB2 and ACTB were used as negative controls. Results were pooled and averaged and are denoted as NSB (NSB = non-specific binding). The level of non-specific occupancy for the H1299 samples is marked as indicated by the dashed line. Plotted are the average occupancy levels expressed as percentage of total input signals. Error bars represent the standard errors of three technical replicates. ^ = *p* < 0.05; * = *p* < 0.01. **B.** Western blotting of endogenous MYC in H1299 and A549 cells. Immuno-reactive bands are representative for one of two independent biological replicates. GAPDH protein detection was used as loading control. Numbers above the panels indicate the relative amount of MYC protein in the two NSCLC-derived cell lines.

To further validate candidate genes identified in the microarray study as predictive of direct c-MYC target genes in human, ChIP assays with the lung cancer cell lines A549 and H1299 were performed (Figure 8A). Therefore chromatin sheared samples were incubated with an anti-MYC monoclonal antibody (black bars) or mouse IgG (grey bars) overnight. Enriched DNA fragments were analyzed by qPCR using specific primers located in the promoter regions of putative MYC target genes. Apart from CCND2 and CDK4 which served as positive controls, c-MYC occupancy at promoters of the genes BIRC5, PRC1 and SRM was studied as well. Furthermore, two specific loci within CCNB2 (within exon 9) and ACTB (in the promoter) were used as negative controls and the results were averaged and are denoted by the column marked NSB (= non-specific binding). As depicted in Figure 8A, qPCR evidenced the average MYC occupancy levels to be enriched in the H1299 cell line. Notably, the endogenous c-MYC expression levels differ by > 5-fold (Figure 8B) between the H1299 and A549 cell lines, confirming a direct correlation between the detection of c-MYC binding on target promoters and the endogenous c-MYC protein levels; therefore, in the latter cell line c-MYC occupancy levels were not significant.

### Chromatin-immunoprecipitation sequencing of non-lung cancer human as well as mouse cell lines

ChIP-seq data deposited in the UCSC Genome Browser (http://genome.ucsc.edu/) was retrieved both using hg19- and mm9-based ENCODE dataset. In total, 7 human cell lines, i.e. lymphoplastoid (GM12878), leukemia (K562), embryonic stem cell (H1-hESC), endothelial cell (HUVEC), hepatoma (HepG2), breast cancer (MCF-7), cervical carcinoma (HeLa) and two *murine* cell lines, i.e. B-cell lymphoma (CH12, analog of human GM12878 cell line) and leukemia (MEL, analog of human K562 cell line) were analyzed for c-Myc binding sites in promoter and other genomic regions of the differentially expressed genes that were originally identified in lung tumors of c-Myc transgenic mice. The comparison is based on overlapping promoter sequences for independent experiments. As shown in Supplementary Table S9, ChIP-seq data with human cell lines confirmed c-Myc binding for 100% and 82% of up-and down-regulated genes, respectively. Consistently, c-Myc occupancy was detected respectively for 73% and 52% of the up- and down-regulated genes (Supplementary Table S9). Fortuitously, the EMSA assay data with nuclear extracts of the positive control (HeLa cells) could directly be compared with the ChIP-seq data for HeLa cells deposited in the ENCODE database. The data was in agreement, i.e. strong c-Myc binding sites seen in EMSA assays with nuclear extracts from transgenic mice at gene specific promoter sites were likewise confirmed in ChIP-seq experiments using HeLa cells. As the molecular organization of orthologous promoters between human and mouse genes differs the obtained results imply evolutionary conservation of regulatory elements. Given the considerable agreement between the gene expression, EMSA, ChIP and ChIP-seq data of human and mouse cancer cell lines the identified genes can be considered as candidate genes of oncogenic c-Myc.

### Gene reporter assays

**Figure 9:**
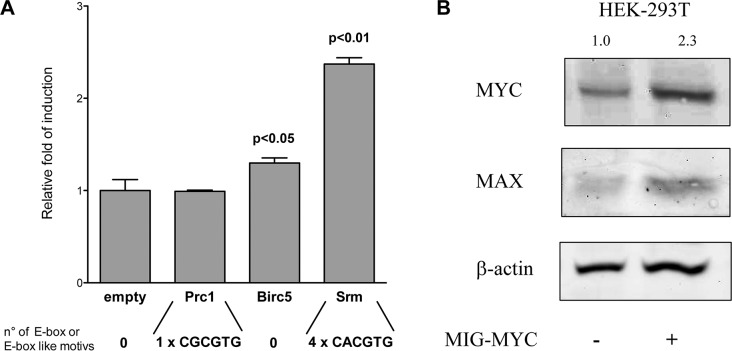
Gene reporter assays and Western Blot in HEK 293T cells **A.** Dual-luciferase assays in transiently transfected cells with the pCZ-REN-P-LUC retroviral vectors containing the Renilla reporter under control of a constitutive promoter and the Firefly reporter under control of the gene specific promoter of the novel candidate genes. Presented are the average ratios of the fold changes of the reporter induction obtained in cells with ectopic over-expression of c-Myc compared to control cells with endogenous c-Myc expression. Error bars represent the standard deviation of at least three biological repeats. **B.** The same total protein extracts prepared for the luciferase assays was used to study the expression of c-Myc and Max proteins by Western blotting. β-actin served as loading control and house-keeping protein. The co-transfection of a c-Myc over-expression plasmid is also indicated (MIG-MYC).

To explore the role of c-Myc in the control of gene expression three up-regulated genes were chosen that presented distinct and interesting features in the promoter sequences. Specifically, Prc1 displayed weakly bound c-Myc due to the presence of a non-consensus E box while Birc5 does not contain any E-boxes or E-box-like elements. In contrast, Srm contains E-box motifs and was shown to be a c-Myc responsive gene in different mouse and human cellular systems. Initially, transfection studies were carried out with a mouse lung cancer cell line isolated from lung tumors of a c-Myc/c-Raf transgenic lung cancer model [37]. Unfortunately, the transfection efficiency was too low for the development of gene reporter assays (data not shown). Therefore, gene reporter assays were developed in HEK 293T cells with mouse specific gene reporter constructs at varying c-Myc expression levels. Here, luciferase assays revealed for the mouse specific Srm promoter a 2-fold (*p* < 0.01), for Birc5 a slight but still statistically significant (*p* < 0.05) increase and for the Prc1 promoter no response (Figure 9A). Western blotting evidenced that the transfection with the c-Myc over-expression vector resulted in a 2-fold increase in c-Myc protein levels (Figure 9B). Interestingly, endogenous Max protein levels were also increased by nearly 2 fold in cells transfected with the c-Myc over-expression vector (Figure 8B). Overall, the gene reporter assays appeared to be in good agreement with the EMSA data, in that strong binding and transactivation could be related to the presence of a consensus E-box element. The lack of reporter induction with the Prc1 construct suggests that the c-Myc responsiveness observed at the level of the endogenous gene could dependent on regulatory sequences located outside of the 2kb region examined. The weak induction of the Birc5 promoter in the absence of any recognizable c-Myc-binding site suggests an indirect effect, such as c-Myc-dependent modulation of other transcription factors/cofactors. However, a c-Myc specific occupancy was also evident from ENCODE mouse ChIP-seq data, located in the same genomic region cloned in the reporter vector. Furthermore, in the human orthologous promoter there are two closely spaced E-box like elements, and the relative c-MYC occupancy appeared to be higher for BIRC5 compared to PRC1 in H1299 cells.

## DISCUSSION

c-Myc engages in complex regulatory networks to influence cellular growth, proliferation, metabolism, differentiation and apoptosis and is frequently regulated in cancers. To better understand c-Myc's role in lung cancer a transgenic disease model was investigated and by use of the surfactant protein C promoter targeted expression of *c-Myc* to respiratory epithelium was achieved to result in induced transcript and protein expression of this oncogene. The subsequent histopathology, genomic, bioinformatics and molecular biology studies helped defining genetic events associated with precancerous lesions and growth of invasive adenocarcinoma and the findings provide opportunities for the development of molecularly targeted therapies.

Specifically, the histopathology of transgenic lung parenchyma evidenced hyper-chromatic precancerous monolayers of atypical bronchiolar alveolar cells. This condition is typical for non-mucinous AHH, and was previously designated as bronchiolo-alveolar carcinoma (BAC), also known as carcinoma *in-situ* or intraepithelial neoplasia. By definition, the AHH of the BAC-type is a non-invasive lesion and characterized by its lepidic growth pattern. The numerous AHH foci are testimony of a dominant intraepithelial growth pattern. Occasionally, AAH was associated with adenocarcinomas and when established the resulting PLACs became aggressive tumors with metastatic spread (Figure 1F). The growth of the invasive PLAC was not hindered by the surrounding non-invasive AHH formations. All invasive tumors were invariably pure PLACs from the very onset of invasion, and the AHH and PLACs are possible sequential events derived from the same ancestral progenitor. Note, the employed transgenic animal model recapitulates the growth behavior observed in human AHH but also recaps pure PLAC tumors.

### c-Myc targeted cell cycle regulators in lung cancer

Under physiological conditions c-Myc activity is tightly regulated [38] with little expression of the protein in resting cells. Upon mitogenic signaling by growth factors and other cell cycle regulators c-Myc expression increases rapidly, nonetheless returns to the basal quiescent state in resting daughter cells. Forced activation of c-Myc allows cells to enter the S-phase and to undergo mitosis in the absence of external factors [39] as described below.

### G1/S promotion

To endorse cell cycle progression a cyclin dependent D1, CdK4 and PCNA complex is formed. Subsequently the Rb/E2F complex is inactivated by phosphorylation of the retinoblastoma protein and the hyper-phosphorylated Rb protein releases the E2F transcription factor which is required for activation of S-phase genes for entry into DNA replication. With c-Myc transgenic lung tumors Cdk4 was significantly up-regulated while expression of one of its inhibitors, i.e. Cdkn2d (p19) was repressed. Additionally, the expression of cyclin D1 was significantly up-regulated and similar results were reported for human lung cancer. Likewise, expression of the transcription factor Dp1 (Tfdp1), i.e. a heterodimeric partner of E2Fs was significantly increased and the findings suggest c-Myc to abrogate the Rb protein/E2F regulatory pathway in the control of G1 cell cycle progression. It is a fascinating aspect of c-Myc biology that it exerts opposite effect on cell cycle regulation with continued activation of c-Myc to promote cellular growth and differentiation, DNA endoreplication and polyploidy. In this regard, it was shown earlier that c-Myc facilitates DNA endoreplication in the absence of cell division by the modulation of cyclin-dependent kinase activity in keratinocytes [40].

### G2/M promotion

The M-phase of the cell cycle is triggered by the cyclin B-Cdk1 (Cdc2a) kinase and with c-Myc transgenic lung tumors a significant up-regulation of cyclin B1 & B2 and associated kinase cdc2a (cdk1) was observed to promote transition into G2/M. Additionally, over-expression of several genes essential for progression of mitosis was identified and included the serine/threonine kinases Nek6 and Stk6 (Aurora-A kinase), Cks1 (cdc28 protein kinase), Cks2 (cdc28 protein kinase regulator subunit 2), cdc20, regulators of cytokinesis Prk1 (protein 1) as well as kinesin family members. The latter codes for molecular motors involved in various kinds of spindle dynamics. Undue activation of Aurora-A kinase, observed at the highest level in small-sized lung tumors causes inappropriate entry into the anaphase leading to mitotic abnormalities and genomic instability and it was shown earlier that Aurora-A kinase amplification overrides the mitotic spindle assembly checkpoint to infer resistance to spindle poisons such as taxol [41]. The observed up-regulation of the proto-oncogene ect2 that plays a critical role in cytokinesis [42] and repression of the tumor suppressor Lats2, which negatively regulates the cell cycle by controlling G1/S and/or G2/M transition [43], are important additional changes induced by c-Myc in lung cancer.

Altogether the transcriptional changes of key cell cycle regulator inform on mechanisms of tumorigenesis and when combined with disabled checkpoints provide an understanding of the genomic instability and karyotypic abnormalities frequently observed in c-Myc-over-expressing cells [44, 45].

### Loss of intracellular control of cell division

Unscheduled cell proliferation causes activation of intracellular checkpoint to either arrest cell cycle progression or to induce apoptosis that can also be mediated by c-Myc [6], therefore restricting its activity [46]. Multiple changes to influence apoptosis and cell cycle programs were observed in lung tumors of c-Myc transgenic mice and included a dramatic repression of the transcription factor Klf-4. This protein is an essential mediator of p53 activity in the control cell cycle progression following DNA damage [47]. P53 regulates expression of several cell-cycle genes in a concerted manner by activating cell-cycle inhibitors and repressing cell-cycle promoters [48]. In lung tumors of c-Myc transgenic mice expression of the transcription factor Hey-1 was lost, and this transcription factor was shown to activate p53 through repression of Mdm-2 transcription and to induce apoptosis *in vivo* [49]. Another transcriptional repressor of Mdm-2 regulated in lung adenocarcinomas was Stat-1, which also binds to p53 and acts as a co-activator to induce p53-responsive genes [50]. Importantly, expression of the tumor suppressor Gas-1 was highly significantly repressed and was detected at extremely low levels in 3 out of 10 tumors only. Note, Gas1 protein blocks cell proliferation in a p53-dependent manner [51]. Conversely, up-regulated genes in tumors included the known c-Myc target Hspa9a (homologous to human mortalin-2). The gene codes for a heat-shock protein 70 family member and is involved in the cytoplasmic sequestration and inactivation of p53 through its direct binding [52]; its over-expression was already reported for human lung adenocarcinomas [53].

### Repression of the intrinsic apoptotic machinery

The present study revealed altered expression of genes in the control of the apoptotic machinery and included the strong induction of Birc5 (survivin), i.e. a member of the inhibitor of apoptosis protein (IAP) family known to affect the function of caspases [54]. Conversely, several genes were down-regulated to affect activity of the anti-apoptotic Bcl-2 and included Bnip-2. This protein interacts directly with death-inhibiting Bcl-2 to induce apoptosis in a caspase-dependent mechanism. Likewise, the significant repression of calpain-2 is of considerable interest. This calcium dependent protease cleaves the Bcl-2 protein and plays an important role in the intrinsic apoptotic pathway [55]. Calpain-2 is strongly expressed in the nervous system and implicated in neuronal apoptosis. Its repression in lung tumors implies a wider role of non-caspase proteases in malignancies. Importantly, a highly significant repression of Ddit3 (= Gadd153) was observed in lung tumors of c-Myc transgenic mice. This basic leucine zipper transcription factor of the dimer forming C/EBP family protein is a key regulator of stress response and enhanced oxidant injury. Its pro-apoptotic effect is linked to down-regulation of Bcl-2 [56]. Furthermore, the tumor suppressor Lats2 was down-regulated and reported to induce apoptosis in lung cancer cells through decreased expression of the anti-apoptotic proteins Bcl-2 and Bcl-x(L) and activation of caspase 9 [57]. Moreover, expression of the tumor suppressor Anp32a (= PHAP) was reduced which promotes caspase-9 activation after apoptosome formation [58]. Some of these proteins with a regulatory role in apoptosis have other functions that can also contribute to cell proliferation and motility. For example, Birc5 is not only implicated in anti-apoptotic programs, but likewise stimulates Aurora-B kinase activity in cytokinesis [59], while Lats2 inhibits the cell cycle by controlling different checkpoints. Anp-32 is involved in repression of transcription as part of the inhibitor of histone acetyl-transferases complex [60], and calpain-2 plays a role in cell migration through regulation of membrane activity and morphology [61].

### Regulatory gene networks

An important finding of the present study was an identification of composite modules of co-bound TFs at c-Myc targeted promoters. The genetic algorithm distinguished between normal and oncogenic c-Myc activity and we propose cooperativity with either Klf7 and Gata3 or Elf5 and Cebpα for repressed and up-regulated genes, respectively thus defining molecular rules for transcriptional responses at targeted promoters. c-Myc transcriptional repression of growth arrest genes was the subject of an earlier report [62] and is caused, in part by the limited binding of the heterodimeric Myc-Max protein complex to an initiator element of targeted promoters, but also involves inhibition of Miz-1 and Sp1 activity. Likewise, c-Myc transcriptional repression in response to TGFβ, APC and DNA damage has been the subject of former reviews [63, 64]. While some of the down-regulated genes in lung tumors of c-Myc transgenic mice did not contain c-Myc binding sites (an observation consistent with the reduced number of promoters bound by c-Myc from ENCODE mouse ChIP-seq data), repression of gene transcription by c-Myc may be independent of binding to the E-box motif, for instance through interaction with Miz-1 that recognizes other regulatory sequences [63].

Taken collectively, the analysis of lung tumors defined c-Myc regulatory networks and master regulators to better understand its transforming capacity. Moreover, in the case of up-regulated genes there was 100% and 73% agreement with ChIP-seq data from 7 different human and 2 mouse cell lines, respectively (see Supplementary Table S9) whereas for repressed genes a similar 82% and 52% agreement was obtained. This comparison is based on orthologous gene promoters with different molecular organizations (human versus mouse).

### Regulation of orthologous genes in human malignancies

A number of genes identified in the present study, i.e. Ccnd1, Stk6, Klf-4, Gas1, Hey1 and Stat-1 were reported to be similarly regulated in various human malignancies [41, 48, 65]. Apart from the remarkable agreement in histological phenotype, the c-Myc disease mouse model mimics closely c-MYC events in human lung cancer and included over-expression of CDK4, CDC2A, TFDP1, CCNB1, CDC20, PRC1, CKS1, CKS2 and MKI67 [66, 67] and similar regulation of some related genes, e.g. up-regulation of NEK2, KIF15 and KIF2C and repression of CDKN2B [67]. In addition, over-expression of anti-apoptotic BIRC5 [67, 68] and heat shock protein HSP70 (47) as well as repression of the tumor suppressor LATS2 [43] was reported for human lung cancer. Up-regulation of Cdk4 and down-regulation of pro-apoptotic Bnip2 were likewise detected in chemically induced *murine* lung tumors [69]. These changes in gene expression point to similarities in the development of lung adenocarcinoma in mouse and human malignancies induced by c-Myc.

In conclusion, the present study identified c-Myc targeted cell cycle and apoptosis genes in lung cancer and permitted the construction of disease-associated molecular circuitries. Some of the identified genes and their coded proteins are likely candidates for the development of molecularly targeted therapies.

## MATERIALS AND METHODS

### Ethics statement

All animal work followed strictly the Public Health Service (PHS) Policy on Humane Care and Use of Laboratory Animals of the National Institutes of Health, USA. Formal approval to carry out animal studies was granted by the animal welfare ethics committee of the State of Lower Saxony, Germany (‘Lower Saxony State Office for Consumer Protection and Food Safety’, LAVES). The approval ID is Az: 33.9-42502-04-06/1204.

### Maintenance of the transgenic mouse line

The development of the SPC/myc-transgenic disease model was previously reported [70]. mice were maintained as hemizygous in the CD2F1-(DBA/2xBalb/C) background and the presence of the transgene was verified by PCR with DNA extracted from tail biopsies using the Platinum PCR SuperMix (Invitrogen, Life technologies, Karlsruhe, Germany) and the following primer pair: 5′-CAGGGCCAAGGGCCCTTGGGGGCTCTCACAG, 3′-GGACAGGGGCGGGGTGGGAAGCAGCTCG.

### Sample collection and preparation

A total of *n* = 19 animals were studied and consisted of *n* = 4 non-transgenic wild type controls, *n* = 5 transgenic and *n* = 10 transgenic lung tumor bearing mice. Note, transgenic animals at the age of about 6–7 month are characterized by an alveolar lining of dysplastic epithelium with no evidence for basement membrane fragmentation and are therefore considered to be epithelial precursor lesions. The lungs of healthy non-transgenic control animals are compared with the non-carcinomatous parenchyma of transgenic lungs and transgenic lung tumors of animals aged 9–13 month.

Lung tumors of individual animals were inspected macroscopically, separated from the surrounding lung tissue and frozen immediately in liquid nitrogen. The tumors were divided into groups according to size in diameter, i.e. 1 mm, 5 mm and > 10 mm. Because of low yield in RNA small tumors (1 mm) dissected from the lungs of a single animal were pooled. Thus, *n* = 3 pools from *n* = 3 individual animals were analyzed. Likewise, tumors of medium and large size obtained from *n* = 4 and *n* = 3 individual animals were studied. In all, 4-non-transgenic wild type controls, 5 transgenic non-carcinomatous and 10 transgenic lung tumor bearing mice were analyzed.

### Histology

The lungs were excised and rinsed with PBS, fixed in 4% PBS-buffered formaldehyde and processed for paraffin embedding using standard operating procedures. Five-μm thick serial sections were stained with hematoxylin and eosin (H and E), hematoxylin only (H) and PAS for light microscopic evaluation.

### Isolation of RNA, production of cRNA, array hybridization and scanning

Total RNA was isolated with the Qiagen RNA purification kit according to the manufacturer's instructions (Qiagen, Hilden, Germany).

The cRNA samples were prepared following the Affymetrix Gene Chip^®^ Expression Analysis Technical Manual (Santa Clara, CA). 10 μg of biotinylated fragmented cRNA were hybridized to the Affymetrix Murine Genome U74v2 GeneChip^®^ expression oligonucleotide array, washed and scanned according to the manufactures’ instructions and described as previously [36].

### Gene expression studies by RT-PCR

The primer 3 software [http://frodo.wi.mit.edu/primer3/input.htm] was used to design intron spanning primers. A description of the genes analyzed and experimental conditions are given in Supplementary Table S2.

### Microarray data analysis

Data analysis was done as recommended by the manufactures and as described in [36]. Initially, the microarray data were processed with the Affymetrix GCOS Software using default settings for scaling or per-chip normalization to generate CEL files. Here the target signal was set to 250 and a microarray quality control report was obtained and the probe set information was converted to 9892 ENSEMBL annotated genes. The gene expression data were further analyzed using different software applications (see also bioinformatics data analysis described below) and the MAS 5.0 algorithm was used to determine statistical significance for expression calls (“Present” or “Absent”). Between groups comparisons (control versus tumor) were carried out as signal logarithm ratio (log2ratio) and a change call (“Increase” or “Decrease”) for the expression level of a given gene. Data from replicate samples were evaluated and compared with the Affymetrix^®^ Data Mining Tool 2 (DMT-2). For each gene mean fold change values were calculated as the ratio of the average expression levels between two groups. Statistical significance was determined by the unpaired two-sided *T*-Test with the *p*-value cut-off set at 0.05. Additionally, the concordance between “Increase” or “Decrease” calls within study groups was determined using the following criteria FC ≥ 3, *p*-value in *T*-test ≤ 0.05, and 100% “Increase” or “Decrease” calls in comparative ranking analysis. Note, in the comparison control healthy versus non-tumor transgenic lungs the filtering criteria were set to FC > 2.5, *p*-value < 0.05 and a changed call of > = 87.5%.

CEL files were also exported from GCOS and uploaded into the ArrayTrack software (National Center for Toxicological Research (NCTR), Food and Drug Administration (FDA), Jefferson, USA) and processed using Total Intensity Normalization after subtracting backgrounds for data management and analysis. With the ArrayTrack software ANOVA, *T*-test and SAM were performed and include adjustment for false discovery rate (FDR) of significantly regulated genes. An FDR of 0.05 was chosen as cut-off for statistical significance.

The microarray data were uploaded to Gene Expression Omnibus (http://www.ncbi.nlm.nih.gov/geo/) with the accession number GSE54829.

### Bioinformatic search for c-Myc binding sites, construction of gene-regulatory networks and composite modules in differentially expressed genes in transgenic non-cancerous and tumor lungs

The cel.files were uploaded onto the geneXplain platform (http://genexplain.com/genexplain-platform-1 version 2.4) and processed using the affy-Bioconductor package software (http://www.bioconductor.org/). After data normalization statistical significance for differentially expressed genes (DEGs) was calculated by performing a hyper-geometric test.

The NO- or background data were defined by removing all DEGs using the default criteria log (base2) fold change > 0.5 and -log (base10) *p*-value > 3 & log (base2) fold change < −0.5 and -log (base10) *p*-value < −3. Such filtering of data yielded 6792 genes. Subsequently, genes with c-Myc binding sites were removed by examining gene specific promoter sequences using 8 different c-Myc position weight matrices (PWMs) deposited in the TRANSFAC(R) 2012.3 ‘vertebrate mouse p0.0001′ database (see Supplementary Table S3). Eventually, 1946 genes (ENSEMBL) devoid of any c-Myc binding sites were defined as NO- or background dataset. In the same way a NO-set for non-tumor transgenic lungs was constructed. Based on the expression of 9437 genes and after normalization and removal of genes with c-Myc binding sites, a total of 2746 genes were considered as NO-set for transgenic non-tumor lungs.

### Experimental or YES-Set data

A filtering criteria of mean FC >= 3.0/<= −3.0, *p*-value in *T*-test <= 0.05 and a present call of 100% were used to define the Yes-set data. In the case of non-tumor transgenic lungs the set filtering criteria yielded 125 up- and 2 down-regulated genes. To obtain more down regulated genes the filtering criteria was slightly relaxed but this did not change the result. Eventually, the filtering criteria were set to a mean FC >= 2.5/<= −2.5, *p*-value in *T*-test <= 0.05 and a present call of > or equal to 87.5%. In all 27, 24 and 233 genes, either up- or down-regulated in tumor or up-regulated in non-tumor transgenic lungs, respectively were identified.

### Search for transcription factor binding sites in differentially expressed genes

The TRANSFAC(R) 2012.3 ‘vertebrate mouse p0.0001′ depository of transcription factor recognition sequences contains nearly 1300 position weight matrices (PWM) and served as reference database. Transcription factor binding sites for up- and down-regulated tumor and up-regulated non-tumor transgenic lungs were defined in the following way: Promoters of annotated genes were interrogated for *cis*-regulatory binding sites of genomic sequences with a length of −1000 to +100 bp relative to TSS. The first ATG codon was considered as tentative TSS (transcription start site). Moreover, the MATCHTM algorithm was used to calculate scores for the matches by use of the so-called information vector. The core and matrix similarity cut-offs for the matrices were used as per TRANSFAC(R) 2012.3 profile ‘vertebrate mouse p0.0001′. The search profile differentiated clearly between the sets of regulated genes and those whose expression was unchanged in lung tumors of c-Myc transgenic mice. In all 32, 28 and 26 different position weight matrices (PWM) with a *p*-value cut-off <= 0.05 for up- and down-regulated tumor and up-regulated non-tumor transgenic experimental datasets were obtained (Supplementary Table S4).

### Co-occupancy of transcription factor binding sites

Composite modules were constructed as previously reported [71] and are based on genetic algorithms to find possible co-occupancy of different transcription factors in co-expressed genes. The underlying multi-component fitness function was previously published [72]. For an initialization of genetic algorithm the parameters were set to 800 iterations, 1000 population size, 800 non-change limit, 50 elite size, 0.25 mutation rate and 0.3 for the penalty rate. Note, in the case of up-regulated tumor associated genes the mutation rate of the genetic algorithm was set to 0.1 to obtain optimal results. A summary of the composite modules is given in Supplementary Table S5. Note, the selection of a specific composite module is based on the number of co-occupied transcription factor binding sites common in the promoters of regulated genes as well as the distance between each pair of binding sites (see Supplementary Table S6).

### Identification of master regulatory molecules

Master regulatory gene networks for up- and down-regulated tumor and up-regulated non-tumor transgenic datasets were constructed using the geneXplain workflow. After annotation of input datasets the tool ‘Master regulator finding over GeneWays network’ (http://anya.igsb.anl.gov/Geneways/GeneWays.html) was applied. Specifically, the GeneWays software is used to automatically extract, analyze, visualize and integrate molecular pathway data from the published peer reviewed literature. It is based on document sorting, term identification, term meaning disambiguation, information extraction, ontology, visualization and system integration [73]. The following filtering threshold was used: score cutoff (0.2), search collection (GeneWays hub), maximum radius (4), FDR cutoff (0.05), Z-score cutoff (1.0), Penalty (0.1) and Decay factor (0.1) (Supplementary Table S7).

### EMSA assays

The oligonucleotides were purchased from MWG Biotech (Eurofins, Ebersberg/Muenchen, Germany) and were used as double-stranded ^32^P-labeled probes as previously reported [37]; for sequence information and EMSA conditions see Supplementary Table S8.

Oligonucleotides were annealed at a final concentration of 1 nmol in a buffer containing 20 mM Tris (pH 7.6), 10 mM MgCl_2_ and 50 mM NaCl at 80°C for 10 min and then were cooled slowly to room temperature overnight and stored at 4°C. Annealed oligonucleotides were diluted to 1:10 in Tris-EDTA buffer (1 mM EDTA, 10 mM Tris, pH 8.0) and 4 pmol were labeled using [32P] ATP (Perkin, Elmer, Rodgau-Jügesheim, Germany) and T4 polynucleotide kinase (New England Biolabs GmbH, Frankfurt am Main, Germany). End-labeled probes were separated from unincorporated [32P] ATP with a Microspin G-25 Column (GE Healthcare Europe GmbH, Freiburg, Germany) and eluted into a final volume of 100 μL.

Nuclear extracts from the liver derived from c-Myc transgenic and tumor-bearing mice were prepared. 5–10 μg nuclear extract and 105 cpm labelled oligonucleotides were incubated in binding buffer consisting of 25 mM HEPES (pH 7.6), 5 mM MgCl_2_, 34 mM KCl, 2 mM DTT, 2 mM Pefablock (Roche Diagnostics GmbH, Mannheim, Germany), 2% aprotinin (Sigma-Aldrich Chemie GmbH, Taufkirchen, Germany), 40 ng poly (dl-dC) / μl and 100 ng bovine serum albumin / μl (PAA Laboratories GmbH, Cölbe, Germany). The binding of nuclear protein was allowed for 20 min on ice and free DNA and DNA-protein complexes were resolved on a 6% polyacrylamide gel. Competition studies were done by adding a 100-fold excess of unlabeled wildtype or mutant oligonucleotides to the reaction mix. Gels were blotted to Whatman 3 MM paper, dried under vacuum, exposed to imaging screens (Imaging Screen-K, Bio-Rad Laboratories GmbH, München, Germany) for autoradiography overnight at room temperature and analyzed using a phosphor imaging system (Molecular Imager FX pro plus; Bio-Rad Laboratories GmbH) and the Quantity One Version 4.2.2 software (Bio-Rad Laboratories GmbH).

### Chromatin immunoprecipitation (ChIP) assays

ChIP assays were performed with the human lung cancer cell lines H1299 and A549 as described previously [74]. Briefly, cells were cultured in 150-mm dishes and at a confluence of 80–90% were subjected to 1% formaldehyde to cross-link proteins with DNA. After 10 min the reaction was stopped by addition of 0.125 M Glycine. Cells were washed twice, harvested and lysed using a SDS-containing lysis buffer. By gently shaking lysates were incubated for 10 min and nuclei were collected by centrifugation at 800 g at 4°C for 5 min. Pellets were re-suspended in buffer containing 0.25% SDS, 200 mM NaCl, 0.1 mg/ml of sonicated salmon sperm DNA and 1X PI (1X Protease Inhibitor, Complete EDTA-free, Roche, Milan, Italy) and were sonicated to shear DNA to fragments ranging between 150 and 400 base pairs (bp) using a Q700 sonicator with a plate horn (QSonica, Newtown, CT, USA). After dilution in ChIP dilution buffer (16.7 mM Tris, 0.01% SDS, 1.1% Triton X-100, 1.2 mM EDTA, 167 mM NaCl), Protein G magnetic Dynabeads (Invitrogen, Life Technologies, Milan, Italy) were added and IPs were carried out overnight at 4°C in a Nutator mixer using 1 μg of anti-MYC monoclonal antibody (clone #: 9E10, Santa Cruz Biotechnology, Milan, Italy) or 1 μg of mouse IgG as a negative control. Immuno-complexes were subsequently washed six times using a magnet as previously indicated DNA was eluted in a two- step process using TE with 1% SDS and TE with 0.67% SDS. The cross-links were reversed overnight at 65°C. RNase A was added and incubated at 37°C for 30 min, followed by Proteinase K treatment for 2 h at 56°C. DNA was purified with the QIAquick PCR purification kit according to manufacturer's recommendation (Qiagen, Milan, Italy). Immunoprecipitated DNA was analyzed for MYC occupancy on selected chromosomal regions surrounding the predicted E- or E-like-boxes by quantitative PCR (qPCR) and enrichment of MYC binding was calculated as percentage of Input DNA (collected before immunoprecipitation) using the ΔC_t_ method. qPCR was performed with the KAPA SYBR Green Universal qPCR mix (Kapa Biosystems, Resnova, Rome, Italy) and all primers were checked for specificity (2% agarose gel) and amplification efficiency (Standard curves with serial dilutions of template genomic DNA). Two different DNA loci were used as ChIP negative controls (β-actin promoter region and CCNB2 exon 9 region). Sequences of primers are available upon request.

### ChIP-seq data retrieval from the UCSC Genome Browser

c-Myc ChIP-seq data from human as well as mouse cell lines deposited in the UCSC Genome Browser (http://genome.ucsc.edu/) was retrieved as follows: first, the track was customized to retrieve all the ChIP-seq data available as part of the encyclopedia for DNA elements (ENCODE) consortium (Version hg19 or mm9 for human or mouse data, respectively). Then, data from 8 different experiments in human cells and 2 from *murine* cells were analyzed by searching for c-Myc binding sites in promoter and other genomic sequences using the gene symbol of the differentially expressed genes identified in tumors of c-Myc transgenic mice.

### Gene reporter assays to examine c-Myc responsiveness of the identified promoter-5′UTR regions

To develop the dual-luciferase vector, we initially cloned the Firefly luciferase cDNA from the commercial plasmid pGL3-basic (Promega, Mannheim, Germany) into the pCZ multi-cloning site using the Hind III and Hpa I restriction endonuclease sites and a standard *in-vitro* ligation procedure. Next, the Renilla control luciferase was PCR amplified along with the constitutive SV40-derived promoter and the poly-A site from the commercial pRL-SV40 plasmid (Promega), sub-cloned in the plasmid pCR4-TOPO (Invitrogen) and then inserted in pCZ upstream of the Firefly luciferase gene using the EcoRI and BamH I sites. This newly constructed vector was named pCZ-REN_LUC. In preparation of the cloning of putative c-Myc target promoters, a linker sequence was introduced between the Renilla and Firefly luciferase cDNA sequences to provide for additional cloning sites (BamHI, Sfi I, Rsr II, Pac I, Cla I, HindIII).

Of note, two gene specific promoters were selected that were identified as regulated based on the microarray data but either did not contain an E-box site (*Birc5*) or contained an E-box like motif (*Prc1*). As positive control the *Srm* promoter was chosen. This known target of c-Myc was also regulated in lung tumors of the present study. Two Kb fragments of the chosen promoters, centered around the TSS and corresponding to the regions that were examined for the presence of c-Myc binding sites by bioinformatics approach, were PCR amplified from mouse genomic DNA with pair of primers containing restriction sites, sub-cloned into the pCR4-TOPO vector and then cloned into pCZ-REN_LUC at the BamHI and Hind III sites (the primers used are available upon request). This vector permits gene reporter assays in transient transfection assay and the development of stable reporter clones by use of the Zeocin selection marker. Correct cloning into pCZ-REN-P-LUC plasmids was confirmed by restriction analysis and DNA sequencing. HEK 293T cells (obtained by ATCC American Type Culture Collection) were seeded onto a 24-well plate (1.2–1.5 × 10^5^) 24 hours prior the transfection to reach almost 90% confluence. The pCZ-REN-P-LUC plasmids (350 ng) were transfected in the HEK cells along with 250 ng of an empty expression vector (MIG-W) or with the c-Myc over-expression plasmid (MIG-MYC, a generous gift of Dr. Alessio Nencioni, Dept. of Internal Medicine, University of Genoa, Italy), using the Lipofectamine 2000 transfection reagent and following the manufacturer's protocol (Invitrogen). 24 hours post-transfection, cells were lysed and dual luciferase assays were conducted using a commercial kit and protocol (Promega) and a multi-label plate reader (Victor3, Perkin Elmer, Nuremberg, Germany). Presented in the results are the averages of the relative fold of induction by MYC over-expression and the standard deviation of three biological repeats. An empty pCZ-REN_LUC vector was used as negative control.

### Western blotting experiments

Proteins from lung tumors of SPC/c-Myc-transgenic mice and/or non-transgenic animals were extracted by sonication in 500 μl benzonase containing 2D-loading buffer and stored at −80°C. The protein concentration was determined by the Bradford protein assay according to the manufacturer's recommendations.

75 or 100 μg of total protein extracts were separated on 10% (Hspa9a) or 12.0% SDS-polyacrylamide gel (c-Myc, Nek6) and blotted onto PVDF membranes in 25 mM Tris and 190 mM glycine at 4°C for 2 h (10%, 12% gel) at 350 mA. Specific antibodies to detect anti-c-Myc rabbit polyclonal (1:500), anti-GRP75 (Hspa9a) goat polyclonal (1:200) were purchased from Santa Cruz Biotechnology (Heidelberg, Germany) while anti-NEK6 rabbit polyclonal (1:100) was purchased from Abgent (San Diego, CA, USA).

Note, c-Myc and Max were also examined in total protein lysates used for the luciferase assays. Here 40 μg of proteins were loaded on a 10% precast acrylamide gel (Bio-Rad, Milan, Italy). Electrophoresis was performed at 180V constant voltage using a mini-protean apparatus (Bio-Rad), followed by semi-dry protein transfer onto PVDF membrane using the semi-dry i-Blot System (Invitrogen) according to the manufacturer's protocol. Immunodetection was performed using primary antibodies directed against c-Myc, Max, GAPDH and β-actin (clone #: N-262, C-124, 6C5 and I-19, respectively, Santa Cruz Biotechnology). Antigen-antibody complexes were visualized using the ECL detection system NEN Life Science Products (PerkinElmer Life Science, Rodgau-Juegesheim, Germany) or ECL plus kit (GE-Healthcare, Milan, Italy) and a ChemiDoc UVP (Celbio, Milan, Italy) as recommended by the manufacturer and recorded with Kodak IS 440 CF (Kodak, Biostep GmbH, Jahnsdorf, Germany).

## SUPPLEMENTARY MATERIALS FIGURES AND TABLES


















