# Supplementary material for: Decoding c-Myc networks of cell cycle and apoptosis regulated genes in a transgenic mouse model of papillary lung adenocarcinomas
Source: Oncotarget. 2015 Oct 2;6(31):31569–92. doi: 10.18632/oncotarget.5035 (PMC4741625; doi:10.18632/oncotarget.5035)
Supplement: Supplementary file 1 [file oncotarget-06-31569-s001.pdf]

SUPPLEMENTARY FIGURES AND TABLES

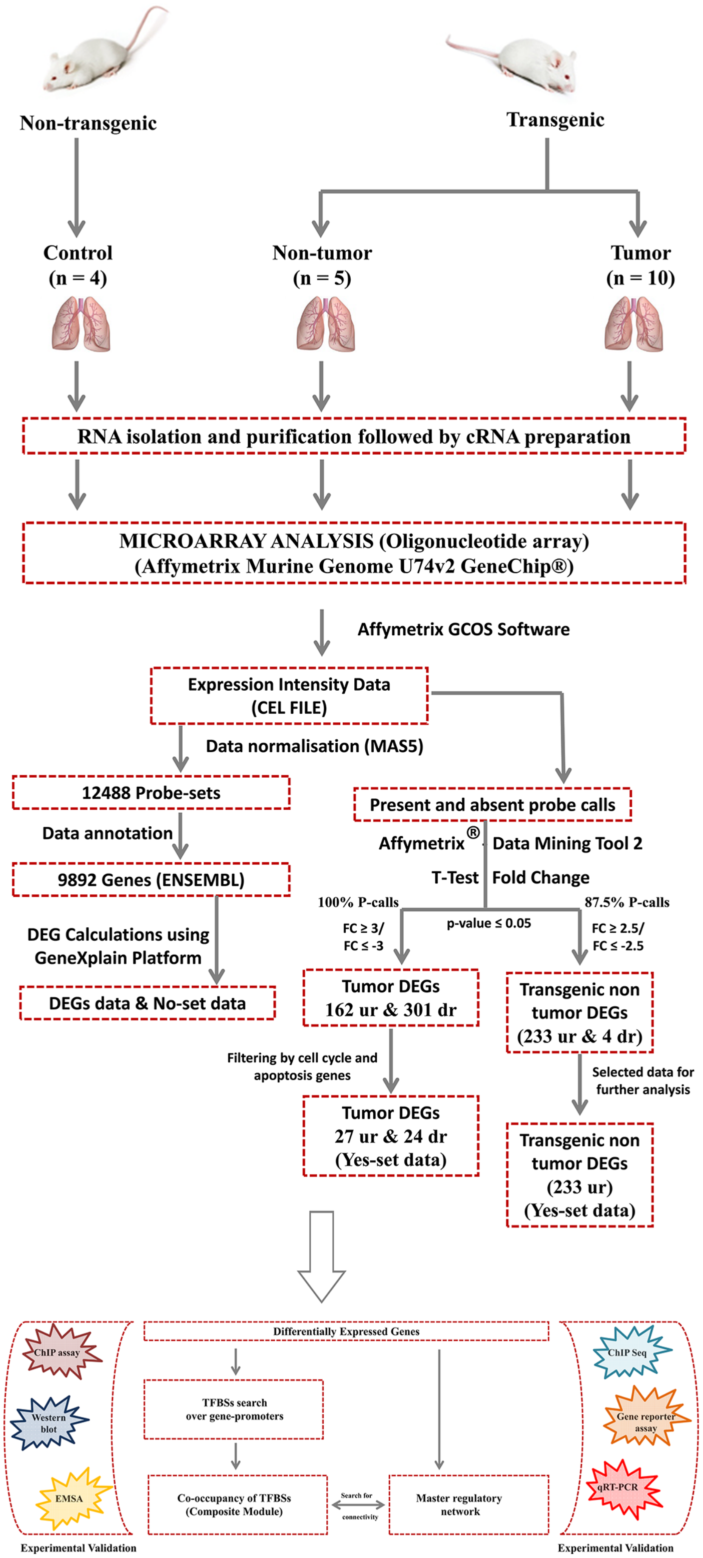

Supplementary Figure S1: Overview of the strategy for data analysis.

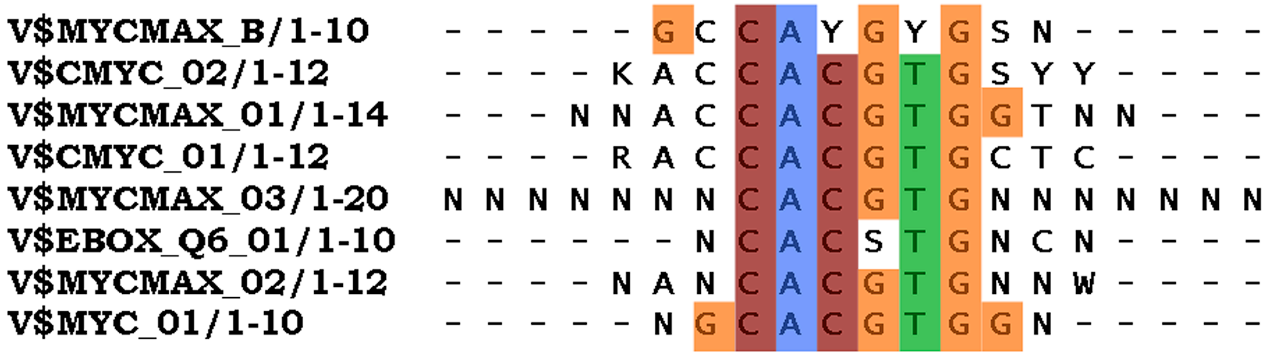

Supplementary Figure S2: Consensus sequence alignment of different position weight matrices for c-Myc. Multiple sequence alignment of eight different PWMs for c-Myc was done with the Clustal-W2 software (http://www.ebi.ac.uk/Tools/msa/clustalw2/). The consensus sequences were taken from TRANSFAC database.

Supplementary Table S1: Gene expression signatures in c-Myc-induced lung papillary adenocarcinoma: genes involved in cell cycle and apoptosis: (A) up-regulated genes; (B) down-regulated genes.

Supplementary Table S2: RT-PCR of selected genes

Gene	Forward Primer	Reverse Primer	Temp (°C)	Cycles
Ccnb1	CAGTTGTGTGCCCAAGAAGA	TCCATTCACCGTTGTCAAGA	57	32
Cdc2a	CTCGGCTCGTTACTCCACTCGA-GCATCAAGAAAGAGGTCAAAGG	CCATTTTGCCAGAGATTCGT	56	34
Stk6	GCCCACTAGGAAAAGGGAAG	CGTTTGCCAACTCAGTGATG	56	34
Cdk4	AACTGATCGGGACATCAAGG	CACGGGTGTTGCGTATGTA	58	30
Nek6	TTGAGATGATGGATGCCAAA	AGCTGTGATGAACACGTTGG	56	32
Prc1	CATGATGCCGAGATTGTACG	CAGCCGATGTAATTCCCACT	57	32
Birc5	GAATCCTGCGTTTGAGTCGT	CAGGGGAGTGCTTTCTATGC	56	38
Ddit3	CTGCCTTTCACCTTGGAGAC	GGGCACTGACCACTCTGTTT	58	34
Satb1	GTGATGGCTCAGTTGCTGAA	CATAGCCCGAAGGTTTACCA	57	32
Hey1	GAGACCATCGAGGTGGAAAA	ACCCCAAACTCCGATAGTCC	58	32
ß-actin	GGCATTGTTACCAACTGGGACG	CTCTTTGATGTCACGCACGATTTC	65	23

Total RNA was isolated with the Qiagen RNA purification kit according to the manufacturer's instructions. Reverse transcription was carried out using Omniscript (Qiagen, Hilden, Germany), Oligo-dT primers (Invitrogen, Karlsruhe, Germany), and RNasin (Promega, Mannheim, Germany), followed by PCR amplification was done with primer pairs shown in the table. ß-actin was used as a housekeeping gene because of its equal expression in all lung samples. PCR reactions were done using Taq Platinum PCR Super-Mix Kit (Invitrogen, Karlsruhe, Germany).

Supplementary Table S3: Search for c-Myc binding sites in regulated genes

Name	Matrix Name	Cutoff	Core Cutoff	Core Start	Core Length	Matrix Logo
V$CMYC_01	V$CMYC_01	0.83038	0.68621	3	5	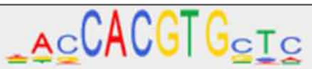
V$CMYC_02	V$CMYC_02	0.8486	0.72931	3	5	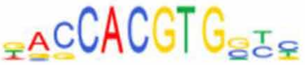
V$EBOX_Q6_01	V$EBOX_Q6_01	0.94214	0.91367	1	5	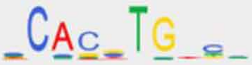
V$MYCMAX_01	V$MYCMAX_01	0.82621	0.69661	4	5	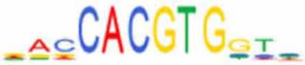
V$MYCMAX_02	V$MYCMAX_02	0.90244	0.84472	3	5	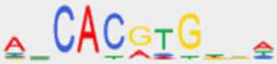
V$MYCMAX_03	V$MYCMAX_03	0.89662	0.84242	7	5	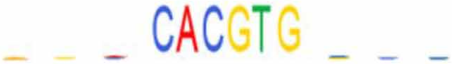
V$MYCMAX_B	V$MYCMAX_B	0.92053	0.86834	1	5	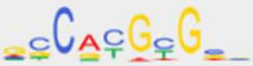
V$MYC_01	V$MYC_01	0.8985	0.84071	2	5	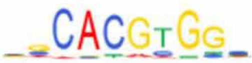

The TRANSFAC(R) 2012.3 'vertebrate mouse p0.0001' depository of transcription factor recognition sequences contains nearly 1300 position weight matrices (PWM) and served as reference database. Eight different PWMs were selected. Each PWM is mentioned with matrix cutoff, core cutoff, core start, core length and matrix logo information.

Supplementary Table S4: Site search result summary for regulated gene datasets. The TRANSFAC(R) 2012.3 'vertebrate mouse p0.0001' depository of transcription factor recognition sequences served as reference database. Transcription factor binding sites for up- and down-regulated tumor and up-regulated non-tumor transgenic experimental datasets were defined in the following way: Promoters of annotated genes were interrogated for cis-regulatory binding sites of genomic sequences with a length of –1000 to +100 bp relative to TSS. The first ATG codon was considered as tentative TSS (transcription start site). Each significant PWM ($p < 0.05$) is mentioned with Yes density per 1000 bp, No density per 1000 bp and Yes-No ratio values.

Supplementary Table S5: Composite module finder for regulated genes in PLAC and non-tumor transgenic lungs. Composite modules were constructed as previously reported [58] and are based on genetic algorithms to find possible co-occupancy of different transcription factors in co-expressed genes. The underlying multicomponent fitness function was previously published [59]. In the present study the parameters were set to 800 iterations, 1000 population size, 800 non-change limit, 50 elite size, 0.25 mutation rate (0.1 for upregulated tumor data) and 0.3 for the penalty rate. Each composite module mentioned with related models, its score and construction parameters are given.

Supplementary Table S6: Frequency of transcription factor binding sites in promoters of regulated genes. The frequency of TF-binding sites is calculated for the various composite modules determined in PLAC (Up or down regulated genes) and non-tumor transgenic lungs. The TF-binding sites search is based on 50 the Transfac(R) 2012.3 'vertebrate mouse p0.0001' profile; promoter sequences with a length of –1000 to +100 bp relative to TSS are considered.

Supplementary Table S7: Master regulatory gene networks in PLAC and non-tumor transgenic lungs. A summary of the gene networks are given along with the network score, FDR, z-score and the genes contributing to the network.

Supplementary Table S8: Oligonucleotide probes for EMSA assays

c-Myc_positive control_BS_WT_F_R	5′-GGAAGCAGACCACGTGGTCTGCTTCC
c-Myc_positive control_BS_Mut_F	5′-GGAAGCAGACCACGGAGTCTGCTTCC
c-Myc_positive control_BS_Mut_R	5′-GGAAGCAGACTCCGTGGTCTGCTTCC
Mouse_Ccnd1_BS 1_F	5′-GAATTTTACACGTGTTGATGAAA
Mouse_Ccnd1_BS 1_R	5′-TTTCATCAACACGTGTAAAATTC
Mouse_Prc1_BS 1_F	5′-CGTCCCGCGCGTGGCAAGTGGAG
Mouse_Prc1_BS 1_R	5′-CTCCACTTGCCACGCGCGGGACG
Mouse_Kif11_BS 1_F	5′-GTTAAAAACACGTGTTGAAGTGA
Mouse_Kif11_BS 1_R	5′-TCACTTCAACACGTGTTTTTAAC
Mouse_Hspa9a_BS 1_F	5′-GGACAACGCCCACGCGCCTGCCA
Mouse_Hspa9a_BS 1_R	5′-TGGCAGGCGCGTGGGCGTTGTCC
Mouse_Trp53_BS 1_F	5′-TCCCCTCCCACGTGCTCACCCTG
Mouse_Trp53_BS 1_R	5′-CAGGGTGAGCACGTGGGAGGGGA
Mouse_Elf5_BS 1_F	5′-GGCTGTTACACGTGCTCATCCAC
Mouse_Elf5_BS 1_R	5′-GTGGATGAGCACGTGTAACAGCC
Mouse_Fasn_BS 1_F	5′-GTCCCCCCGCGTGGCCCTGGTGT
Mouse_Fasn_BS 1_R	5′-ACACCAGGGCCACGCGGGGGGAC
Mouse_Srm_BS 3_F	5′-TCGCCTGCCACGTGTCACCCCGA
Mouse_Srm_BS 3_R	5′-TCGGGGTGACACGTGGCAGGCGA
Mouse_Lats2_BS 1_F	5′-AGGAGGGGTCACGTGACGCCCGT
Mouse_Lats2_BS 1_R	5′-ACGGGCGTCACGTGACCCCTCCT
Mouse_Cebpa_BS 1_F	5′-ACCACGGACCACGTGTGTGCGGG
Mouse_Cebpa_BS 1_R	5′-CCCGCACACACGTGGTCCGTGGT
Mouse_Cebpa_BS 2_F	5′-ACAGCGGCGCCACGCGCAGGCTG
Mouse_Cebpa_BS 2_R	5′-CAGCCTGCGCGTGGCGCCGCTGT
Mouse_Foxf1a_BS 1_F	5′-CCTGATGCGCGTGGCCTCCCGCA
Mouse_Foxf1a_BS 1_R	5′-TGCGGGAGGCCACGCGCATCAGG
Mouse_Tbx3_BS 2_F	5′-GTCTCTGCACGTGGCTGCGGGTG
Mouse_Tbx3_BS 2_R	5′-CACCCGCAGCCACGTGCAGAGAC
Mouse_Klf7_BS 1_F	5′-CTTCCTGATCCACGCGCTGGAGT
Mouse_Klf7_BS 1_R	5′-ACTCCAGCGCGTGGATCAGGAAG

Supplementary Table S9: Publically available ChIP-seq data. c-Myc ChIP-seq data deposited in the UCSC Genome Browser (http://genome.ucsc.edu/) was retrieved as follow: first, the track was customized to retrieve all the ChIP-seq data available as part of the encyclopedia for DNA elements (ENCODE) consortium (Version hg19 and mm9 for *human* and *mouse* data, respectively). Then, data from 8 different experiments in *human* cells (7 cell lines) and 2 from *murine* cells were analyzed by searching for c-Myc binding sites in promoter and other genomic sequences using the gene symbol of the differentially expressed genes identified in tumors of c-Myc transgenic *mice*.

Supplementary Table S10: Differential gene expression in non-tumor transgenic lung. Microarray data of control-wild type lung tissue extracts were compared with non-tumor transgenic lungs. Next to p-values the number of calls at the probe level for individual genes is given. Note, a total of 16 probe pairs per sequence is the maximum call for an individual gene in this particular experiment for either up or down regulated genes.

Supplementary Table S11: Frequency of c-Myc binding motifs at gene specific promoter sites of regulated genes. A total of 8 different PWM derived from the TRANSFAC(R) 2012.3 ‘vertebrate mouse p0.0001’ depository of transcription factor recognition was used for data analysis.
